# AIFM2 promotes hepatocellular carcinoma metastasis by enhancing mitochondrial biogenesis through activation of SIRT1/PGC-1α signaling

**DOI:** 10.1038/s41389-023-00491-1

**Published:** 2023-09-21

**Authors:** Sanxing Guo, Fengying Li, Yixuan Liang, Yufei Zheng, Yingyi Mo, Deyao Zhao, Zhixiong Jiang, Mengmeng Cui, Lixia Qi, Jiaxing Chen, Lixin Wan, Guoyong Chen, Sidong Wei, Qi Yang, Junqi Liu

**Affiliations:** 1 Department of Oncology, The First Affiliated Hospital of Zhengzhou University, 450052 Zhengzhou, Henan China; 2 Department of Radiation Oncology, The First Affiliated Hospital of Zhengzhou University, 450052 Zhengzhou, Henan China; 3 Laboratory of Cancer Biomarkers and Liquid Biopsy, School of Pharmacy, Henan University, 475004 Kaifeng, Henan China; 4 Department of Hepatobiliary Pancreatic Surgery, Henan Provincial People’s Hospital; Zhengzhou University People’s Hospital, Henan University People’s Hospital, 450003 Zhengzhou, Henan China; 5 School of Life Sciences, Henan University, 475004 Kaifeng, Henan China

**Keywords:** Liver cancer, Acetylation

## Abstract

AIFM2 is a crucial NADH oxidase involved in the regulation of cytosolic NAD^+^. However, the role of AIFM2 in the progression of human cancers remains largely unexplored. Here, we elucidated the clinical implications, biological functions, and molecular mechanisms of AIFM2 in hepatocellular carcinoma (HCC). We found that AIFM2 is significantly upregulated in HCC, which is most probably caused by DNA hypomethylation and downregulation of miR-150-5p. High expression of AIFM2 is markedly associated with poor survival in patients with HCC. Knockdown of AIFM2 significantly impaired, while forced expression of AIFM2 enhanced the metastasis of HCC both in vitro and in vivo. Mechanistically, increased mitochondrial biogenesis and oxidative phosphorylation by activation of SIRT1/PGC-1α signaling contributed to the promotion of metastasis by AIFM2 in HCC. In conclusion, AIFM2 upregulation plays a crucial role in the promotion of HCC metastasis by activating SIRT1/PGC-1α signaling, which strongly suggests that AIFM2 could be targeted for the treatment of HCC.

## Introduction

Hepatocellular carcinoma (HCC) is one of the most deadly types of human cancer [1]. If not diagnosed early, the survival rate of patients is quite low, which is largely due to distant metastasis with limited effective therapies [2]. Therefore, elucidating the molecular mechanisms underlying the metastasis of HCC is essential for developing new therapeutic approaches for this deadly malignancy.

Apoptosis-inducing factor mitochondria-associated 2 (AIFM2) has been identified as a suppressor of endogenous ferroptosis [3]. Recently, AIFM2 was also found to act as a crucial NADH oxidase involved in the regulation of cytosolic nicotinamide adenine dinucleotide NAD^+^ [4], which has been well-established as a key biomolecule involved in many critical processes [5]. NAD^+^ has been well-known as a critical regulator of cell metabolism (mainly in oxidative phosphorylation and redox reactions) and signal transduction. Additionally, NAD^+^ also serves as a substrate for multiple enzymes, especially sirtuins, which consist of seven members ranging from SIRT1-7 [6]. Among them, SIRT1 and SIRT2 are mainly located in the cytoplasm, the others are mainly located in the nucleus (SIRT6 and SIRT7) or mitochondria (SIRT3, SIRT4, and SIRT5) [7]. Accumulating evidence has revealed that alterations in NAD^+^ homeostasis are closely associated with many age-related diseases, including cancer [8], implying that AIFM2 may play a role in human cancer. However, the role of AIFM2 in regulating the development and progression of human cancers remains largely unexplored, especially in HCC.

In the current study, we aimed to investigate the expression pattern, clinical implication, biological functions, and molecular mechanisms of AIFM2 in hepatocellular carcinoma (HCC).

## Materials and methods

### HCC patient tissue samples and cell lines

Hepatocellular carcinoma tumors and adjacent normal tissues were collected from 243 HCC patients who underwent surgical resection in the First Affiliated Hospital of Zhengzhou University. All patients have signed the written informed consent. This study was approved by the ethical review board of the First Affiliated Hospital of Zhengzhou University. HCC cell lines (SNU-449, Hep3B, HLE, SNU-354, SNU-423, and HLF) and normal hepatocyte LO-2 were obtained from ATCC. All cells were cultured in Dulbecco’s modified Eagle’s medium (DMEM) with 10% FBS in 5% CO_2_ at 37 °C. Cell lines were authenticated by short tandem repeat (STR) analysis and tested to be mycoplasma-free.

### Reagents

MG132, a proteasome inhibitor, was purchased from Cell Signaling Technology (# 2194S). EX-527 (an inhibitor of SIRT1) and resveratrol (an activator of SIRT1) were purchased from Selleck Chemicals (#S1542 and #S1396).

### Knockdown and forced expression of target genes

Two distinct siRNAs synthesized by Genepharma (Shanghai, China) were used to knockdown AIFM2 expression in HCC cell lines. The target sequence of siRNAs were 5’-TGCTATTCTTCTGGAATAAGATG-3’ for si-AIFM2#1 and 5’- AAGAAGAAAGAGCTAGATAAATG-3’ for si-AIFM2#2. The sequence of miR-150-5p was UCUCCCAACCCUUGUACCAGUG. Stable knockdown of AIFM2 or PGC-1α was generated by lentiviral-based shRNA and selected by puromycin. For overexpressing construct, AIFM2 or PGC-1α cDNA was cloned into pcDNA3.1 (Invitrogen, V790-20) vector. Transfections were performed using Lipofectamine 2000 (Invitrogen) following the manufacturer’s instructions.

### Real-time quantitative PCR

Total RNA was extracted from tissues and cell lines using Trizol reagent (ThermoFisher) and cDNA was synthesized using PrimeScript RT reagent Kit (Takara), according to their manufacturer’s guidelines. The resulting cDNA was then used for real-time PCR analysis with SYBR Green PCR Master Mix (Takara). U6 snRNA and β-actin were used for normalizations. The primer sequences were provided in Supplementary Table 1 (Table S1).

### Western blotting

Cell lysates were prepared with RIPA lysis buffer and the protein concentration was determined by BCA assay (Thermo Scientific). Protein was separated in SDS–polyacrylamide gels and transferred onto polyvinylidene fluoride membranes (Millipore). The membranes were blocked in 5% non-fat milk and incubated with specific primary and secondary antibodies. Primary antibodies used are provided in Table S2. The blots were visualized using the enhanced chemiluminescence assay.

### Immunohistochemical (IHC) staining

Formalin-fixed and paraffin-embedded tissue samples were cut into 5 μm thick on polarized glass. Antigen retrieval was performed in a hot citrate buffer (pH = 6.0). The sections were then incubated with 5% goat serum and 3% H_2_O_2_, followed by antibodies at the indicated concentrations provided in Table S2. Nuclei were counterstained with hematoxylin. Images were acquired under a light microscope.

IHC staining scores were calculated by multiplying the positive staining area score and staining intensity score. The positive staining area was scored as 0 (when 0% area was positively stained), 1 point (when 0%–25% area was positively stained), 2 points (when 26%–50% area was positively stained), 3 points (when 51%–75% area was positively stained), and 4 points (when 76%–100% area was positively stained). Staining intensity was scored as 0 points (negative staining), 1 point (weak staining), 2 points (moderate staining), and 3 points (strong staining).

### Cell viability and colony formation assays

Cell viability was determined by MTS assay. Briefly, 5000 cells were seeded into each well of the 96-well plate. MTS working solution was added and cell viability was assessed after 1 h of incubation at 490 nm at the indicated time point. For the colony formation assay, 1000 cells were seeded into a 6-well plate and grown for 12 days. Colonies were stained with 5% crystal violet and their numbers were counted.

### Transwell migration and invasion assays

Transwell migration and matrigel invasion chambers were used for the determination of cell migration and invasion abilities. HCC cells in a serum-free medium were seeded into the upper chamber. A cell culture medium containing 10% FBS was added to the lower chamber. After growing for 24 h (for migration assay) or 48 h (for invasion assay), successfully migrated or invaded cells were stained with 5% crystal violet, and their numbers were counted under a light microscope.

### Flow cytometry analysis for cell cycle and apoptosis

Flow cytometry was used to analyze cell cycle and apoptosis with a cell cycle and apoptosis kit (US Everbright Inc) according to their manufacturer’s instructions, respectively. The results were analyzed using flow cytometry (Beckman).

### In vivo lung metastasis assay

A total of 5 × 10^6^ HCC cells with AIFM2 knocked-down or overexpressed were intravenously injected through the tail vein into 4-week-old male BALB/c nude mice, which were randomly divided into different groups (6 mice per group). Five weeks following cell injection, the mice were sacrificed and their lungs were excised for Hematoxylin-Eosin staining. The number of tumor nodules formed in the lungs was counted under a light microscope. All animal experimental procedures were approved by the animal ethics committee of the First Affiliated Hospital of Zhengzhou University and carried out in accordance with its guidelines.

### Methylation-specific PCR (MSP)

DNA was extracted from tissue samples with a DNA Extraction Kit (ThermoFisher) according to the manufacturer’s guidelines. A Methylation-Gold Kit (Zymo Research Corporation) was used for sodium bisulfite treatment. The primer sequences used for methylated (M) and unmethylated (U) AIFM2 were provided in Table S1.

### Detections of SIRT1 and SIRT2 activities

The activities of SIRT1 and SIRT2 in HCC cells were determined with a fluorometric SIRT1 activity assay kit (Abcam, ab156065) or a fluorometric SIRT2 activity assay kit (Abcam, ab156066) according to their manufacturer’s instructions. Results were normalized to protein concentrations.

### Measurement of intracellular NAD^+^ level

The intracellular NAD^+^ levels were measured with a NAD^+^/NADH quantitation kit (Abcam) as per the manufacturer’s instruction. Results were normalized using protein concentrations.

### Detections of oxygen consumption rate (OCR) and mitochondrial respiratory chain complexes activity

Oxygen consumption rate (OCR) was assessed by the XF96 Extracellular Flux Analyzer (Seahorse Bioscience) following the manufacturer’s protocols. The activities of mitochondrial respiratory chain complexes I to V were determined using a commercial kit (#ab110419) according to the manufacturer’s instructions.

### Detection of membrane potential

Mitochondrial membrane potential was evaluated with JC-1 dye (Beyotime Biotechnology, C2006) following the manufacturer’s instruction. The results were observed with a laser scanning confocal microscopy (Olympus).

### MitoTracker staining

Mitochondrial mass was evaluated by staining with MitoTracker green fluorescent dye (Molecular Probes, M7514) for 30 min at 37 °C. After gently washing three times with PBS, a laser scanning confocal microscopy (Olympus) was used for analysis of the staining results.

### Mitochondrial DNA content detection

A qPCR-based method was used for mitochondrial DNA content detection. Briefly, total DNA was extracted from HCC cells with a DNA Extraction Kit (ThermoFisher) according to the manufacturer’s guidelines. Then, extracted total DNA was used for real-time PCR analysis for the levels of mitochondrial ND1 and nuclear HGB. Relative mitochondrial DNA content was calculated by the ratio of the mitochondrial ND1 gene to nuclear HGB. The primer sequences for ND1 and HGB are provided in Table S1.

### Statistical analysis

All assays were conducted at least three times, and the data are expressed as the mean ± SD. SPSS software (version 18.0) was used to perform the statistical analysis. Comparisons between two or multiple groups were performed by two-tailed student’s *t*-test or one-way ANOVA. Patient survival was analyzed by the Kaplan–Meier method and log-rank test. *p*-value less than 0.05 was considered to indicate a significant difference. Data from human HCC tissue samples, animals, and cell lines were collected in a blinded manner.

## Results

### AIFM2 expression is markedly upregulated in HCC and its upregulation is associated with poor patient survival

**Fig. 1:**
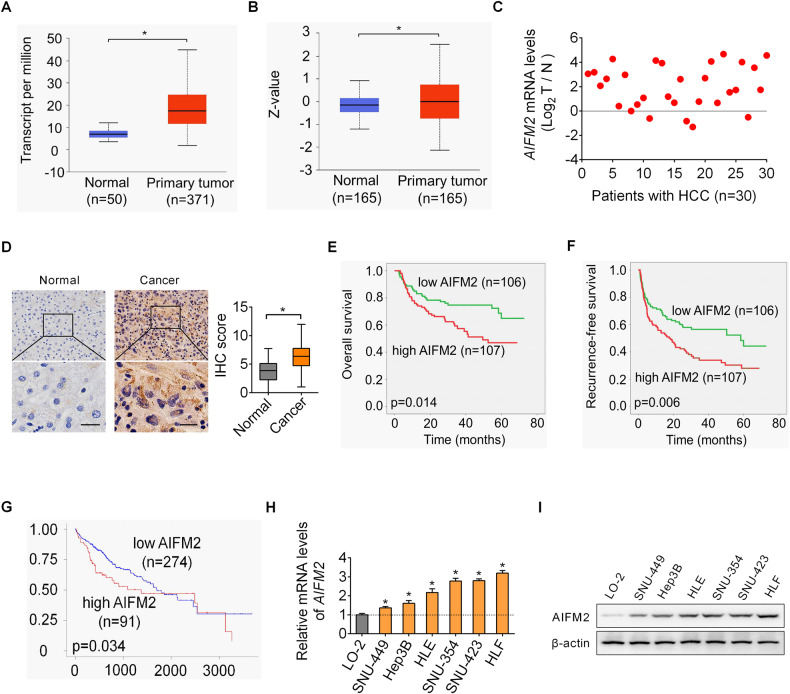
AIFM2 expression is markedly upregulated in HCC and its upregulation is associated with poor patient survival. **A** and **B** The expression of AIFM2 in HCC was assessed using the online UALCAN database at mRNA (**A**) and protein (**B**) levels. **C** AIFM2 expression was determined by qRT-PCR analysis in 30-paired tumor and adjacent non-tumor tissue samples. **D** AIFM2 expression was determined by immunohistochemistry (IHC) analysis in another cohort of 213-paired tumor and adjacent non-tumor tissue samples. Scale bars, 20 μm. **E** and **F** Kaplan–Meier curves of overall survival (**E**) and recurrence-free survival (**F**) in different AIFM2 expression groups. **G** Kaplan–Meier survival analysis in different AIFM2 expression groups using the online UALCAN database. **H** and **I** qRT-PCR and western blot analysis for AIFM2 expression in HCC and normal hepatocyte cell lines.

We first determined the expression of AIFM2 in HCC using the online UALCAN database [9]. A significant upregulation of AIFM2 at both mRNA and protein levels was observed in tumor tissues of HCC as compared with normal liver tissues (Fig. 1A, B). Upregulation of AIFM2 was further confirmed at mRNA level by quantitative reverse transcription PCR (qRT-PCR) analysis in 30-paired tumor and adjacent non-tumor tissue samples (Fig. 1C) and at protein level by immunohistochemistry (IHC) analysis in another cohort of 213-paired tumor and adjacent non-tumor tissue samples (Fig. 1D). Correlation analysis indicated a significant positive correlation between the expression of AIFM2 and clinicopathological feature of tumor metastasis (Table S3), implying that AIFM2 may play an oncogenic role in HCC progression. Kaplan–Meier survival analysis indicated that HCC patients with high AIFM2 expression had a worse overall survival and higher recurrence than patients with low AIFM2 expression (Fig. 1E, F). Similarly, survival analysis using the online UALCAN also revealed that upregulation of AIFM2 is associated with poor survival of HCC patients (Fig. 1G). Moreover, in line with the expression of AIFM2 in tumor tissues of HCC, a significant increase in AIFM2 expression was also observed in HCC cell lines as compared to normal hepatocytes by using qRT-PCR and western blot analysis (Fig. 1H, I).

Additionally, the expression of AFIM2 was also analyzed in several other human cancer types using the online database Sangerbox (http://vip.sangerbox.com/). Similar to HCC, the expressions of AIFM2 are also significantly increased in LUAD (Lung adenocarcinoma), STES (Stomach and Esophageal carcinoma), KIRP (Kidney renal papillary cell carcinoma), STAD (Stomach adenocarcinoma), UCEC (Uterine Corpus Endometrial Carcinoma), KICH (Kidney Chromophobe) and CHOL (Cholangiocarcinoma), indicating that AIFM2 may play a crucial oncogenic role in multiple human cancer types.

### Knockdown of AIFM2 suppressed metastasis of HCC cells in vitro and in vivo

**Fig. 2:**
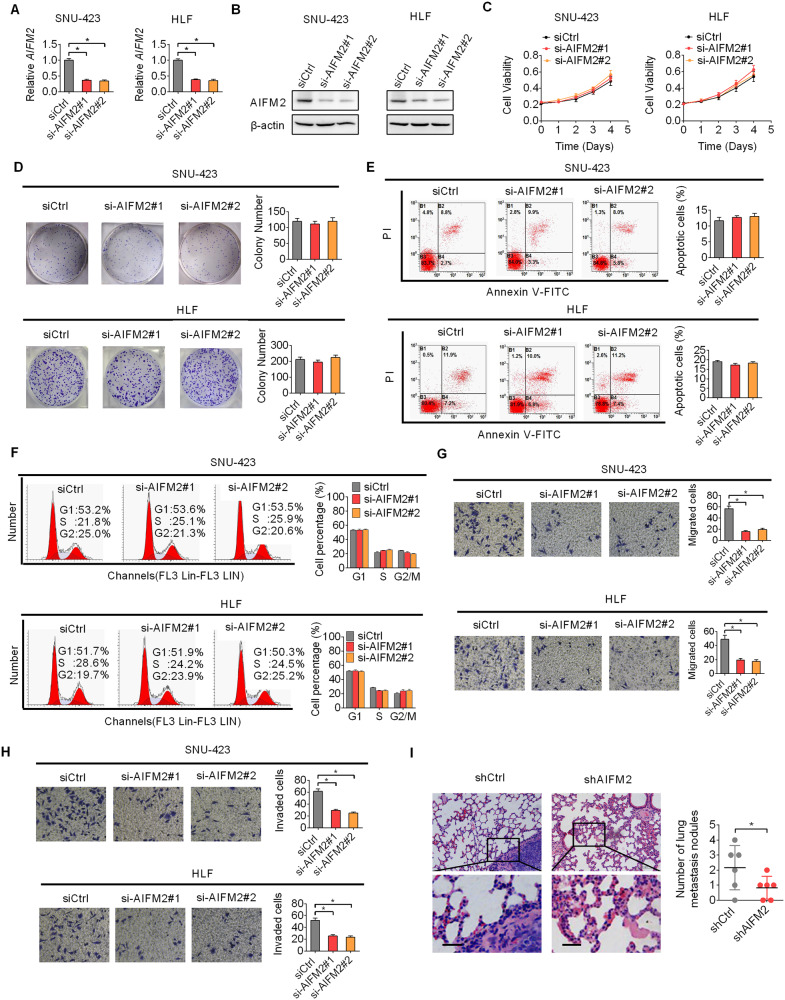
Knockdown of AIFM2 suppressed metastasis of HCC cells in vitro and in vivo. **A** and **B** Downregulation of AIFM2 was tested in SNU-423 and HLF cells upon transfection with siRNAs targeting AIFM2 by qRT-PCR and western blot analysis. **C** and **D** Short- and long-term cell proliferation was determined by MTS cell viability (**C**) and colony formation (**D**) assays in SNU-423 and HLF cells upon transfection with siRNAs targeting AIFM2. **E** and **F** Cell apoptosis and cell cycle were analyzed by flow cytometry in SNU-423 and HLF cells upon transfection with siRNAs targeting AIFM2. **G** and **H** Transwell migration and invasion assays in SNU-423 and HLF cells upon transfection with siRNAs targeting AIFM2. **I** Number of lung metastases was evaluated by Hematoxylin-Eosin staining in nude mice injected with AIFM2 knockdown or control SNU-423 cells (6 mice per group). Scale bars, 20 μm.

Significant upregulation of AIFM2 promoted us to hypothesize that AIFM2 may function as an oncogene in HCC. To determine the functions of AIFM2 in HCC cells, AIFM2 was knocked down in SNU-423 and HLF cells expressing high levels of AIFM2 (as indicated in Fig. 1H, I). Strongly downregulation of AIFM2 in SNU-423 and HLF cells was observed upon transfection with siRNAs targeting AIFM2 (Fig. 2A, B). MTS cell viability and colony formation assays indicated that AIFM2 knockdown had no significant effect on both short- and long-term cell proliferation of HCC cells (Fig. 2C, D). Similarly, no notable changes in cell apoptosis and cell cycle were also observed upon AIFM2 knockdown in SNU-423 and HLF cells (Fig. 2E, F). Next, we determined the effect of AIFM2 knockdown on cell migration and invasion in SNU-423 and HLF cells. The results showed that AIFM2 knockdown led to markedly decreased cell migration and invasion (Fig. 2G, H), as evidenced by transwell migration and invasion assays. To validate the effect of AIFM2 on the metastasis of HCC cells in vivo, AIFM2 stable knockdown SNU-423 cells (Fig. S2A and S2B) were constructed and intravenously injected into nude mice (6 mice per group) through the tail vein. In concordance with the in vitro results, the number of lung metastases was also significantly lower in the AIFM2 knockdown group as compared with the control group (Fig. 2I). Together, these results suggest that AIFM2 plays a crucial role in the promotion of HCC metastasis.

### Forced expression of AIFM2-promoted metastasis of HCC cells in vitro and in vivo

**Fig. 3:**
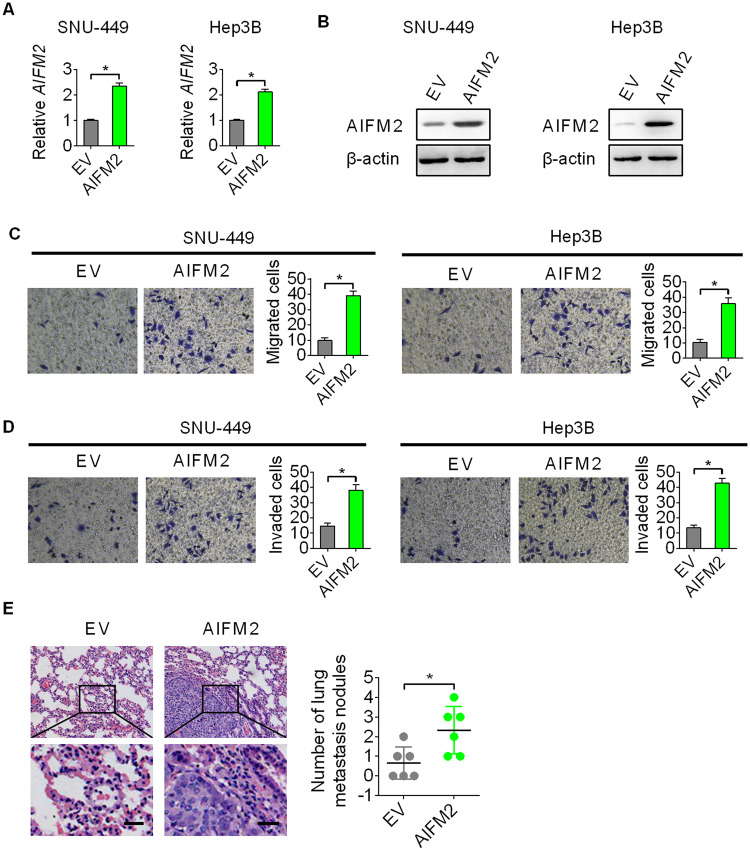
Forced expression of AIFM2-promoted metastasis of HCC cells in vitro and in vivo. **A** and **B** Overexpression of AIFM2 was tested in SNU-449 and Hep3B cells upon transfection with AIFM2 expression vector by qRT-PCR and western blot analysis. **C** and **D** Transwell migration and invasion assays in SNU-449 and Hep3B cells upon transfection with AIFM2 expression vector. **E** Number of lung metastases was evaluated by Hematoxylin-Eosin staining in nude mice injected with AIFM2 overexpression or control SNU-449 cells (6 mice per group). Scale bars, 20 μm.

To provide more evidence for the promotive function of AIFM2 on HCC metastasis, we next overexpressed AIFM2 in SNU-449 and Hep3B cells expressing low levels of AIFM2 (as indicated in Fig. 1H, I). Strongly overexpression of AIFM2 was confirmed by qRT-PCR and upon transfection with AIFM2 expression vector by qRT-PCR and western blot analysis in SNU-449 and Hep3B cells (Fig. 3A, B). Both transwell migration and invasion assays showed that forced expression of AIFM2 significantly enhanced the migration and invasion abilities of SNU-449 and Hep3B cells (Fig. 3C, D). Additionally, forced expression of AIFM2 (Fig. S2C, S2D) also markedly elevated the in vivo metastasis of SNU-449 cells, as indicated by the increased number of lung metastases in nude mice injected with AIFM2 overexpression SNU-449 cells, as compared to those injected with control SNU-449 cells (Fig. 3E).

### Upregulation of AIFM2 is mainly caused by DNA hypomethylation and decreased miR-150-5p expression

**Fig. 4:**
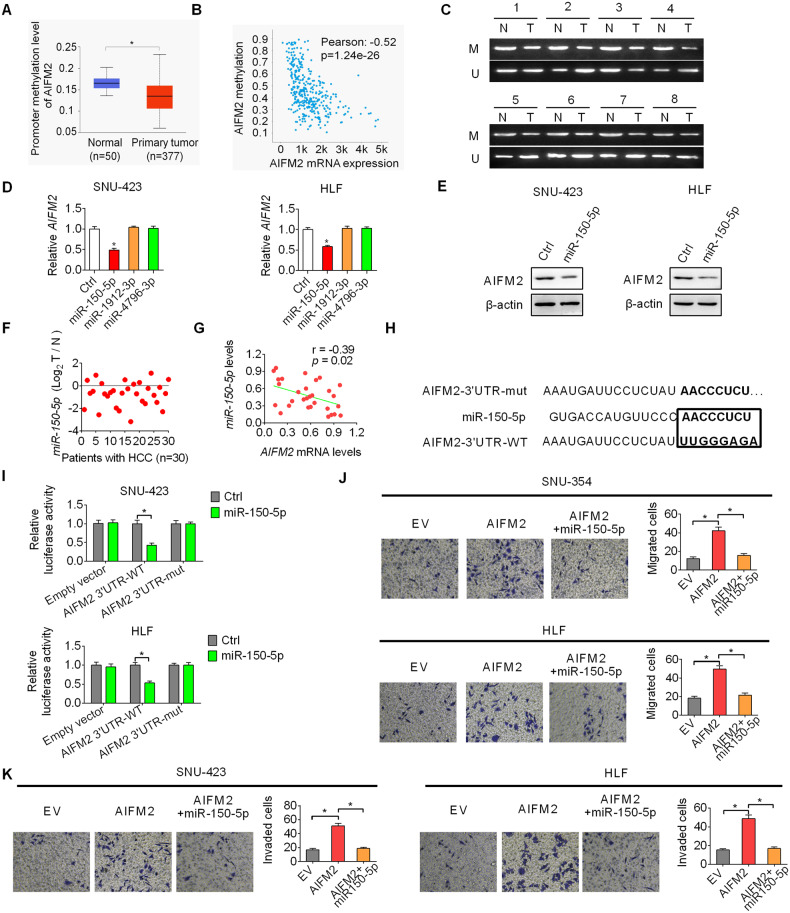
Upregulation of AIFM2 is mainly caused by DNA hypomethylation and decreased miR-150-5p expression. **A** Promoter methylation of AIFM2 in HCC was analyzed using the online UALCAN database. **B** Correlation between the DNA methylation and mRNA expression levels of AIFM2 in HCC was analyzed using the online cBioportal database. **C** Methylation-specific PCR was used to detect the methylation level of AIFM2 in 8-paired tumor and adjacent non-tumor tissues (M, methylated; U, unmethylated). **D** and **E** AIFM2 expression was examined by qRT-PCR and western blot analysis in SNU-423 and HLF cells transfected with indicated miRNAs. **F** miR-150-5p expression was determined by qRT-PCR analysis in 30-paired tumor and adjacent non-tumor tissue samples. **G** Correlation between the expression of AIFM2 and miR-150-5p in HCC tissues (*n* = 30). **H** Wild-type and corresponding mutant type AIFM2 3’-UTR at the binding sites of miR-150-5p. **I** Luciferase assay of AIFM2 3’-UTR and AIFM2 3’-UTR-mut reporters co-transfected with miR-150-5p in HCC cells. **J** and **K** Rescue transwell migration (**G**) and invasion (**H**) experiments were carried out in SNU-449 and Hep3B cells.

Upregulation of AIFM2 in HCC at both mRNA and protein levels suggests that its upregulation may occur at the pre-translational level. UALCAN-based analysis showed a significantly decreased promoter methylation level of AIFM2 in HCC in comparison with normal liver tissues (Fig. 4A). In agreement with this, a significant negative association was also found between the methylation and mRNA expression levels of AIFM2 (Pearson correlation coefficient:-0.52; *p* < 0.001; Fig. 4B) using the online cBioportal database. We also determined the methylation level of AIFM2 in 8-paired tumor and adjacent non-tumor tissues using methylation-specific PCR. We observed obviously decreased methylation level of AIFM2 in tumor tissues of HCC, as compared with non-tumor tissues (Fig. 4C). These results imply that DNA hypomethylation may contribute to the upregulation of AIFM2 in HCC. We also explored the contribution of microRNAs (miRNAs), the well-known post-transcriptional regulators of gene expression, to the upregulation of AIFM2 in HCC using a target prediction platform mirDIP [10]. We found that only transfection with miR-150-5p, which is among the top three predicted miRNAs targeting AIFM2 (Fig. S3), markedly decreased AIFM2 expression at both mRNA and protein levels in SNU-423 and HLF cells (Fig. 4D, E). As expected, the levels of miR-150-5p were markedly decreased in 30-paired tumor tissues of HCC as compared with normal liver tissues (Fig. 4F). Additionally, a significant negative correlation also exists between the expressions of AIFM2 and miR-150-5p in tissues of HCC (Fig. 4G). Next, we performed a luciferase reporter assay to verify the binding between the AIFM2 3’-UTR and miR-150-5p with a mutated or wild-type AIFM2 3’-UTR coupled luciferase reporter (Fig. 4H). As shown in Fig. 4I, the luciferase activity was significantly decreased in HCC cells with wild-type AIFM2 3’-UTR upon miR-150-5p transfection, while no change in the luciferase activity was observed in HCC cells with mutated AIFM2 3’-UTR upon miR-150-5p transfection. Furthermore, transwell migration and invasion assays revealed that miR-150-5p transfection markedly attenuated AIFM2 upregulation-enhanced HCC metastasis (Fig. 4J, K). The above findings suggest that AIFM2 upregulation in HCC can be attributed to DNA hypomethylation and downregulated miR-150-5p expression.

### AIFM2-promoted mitochondrial biogenesis and oxidative phosphorylation in HCC cells

**Fig. 5:**
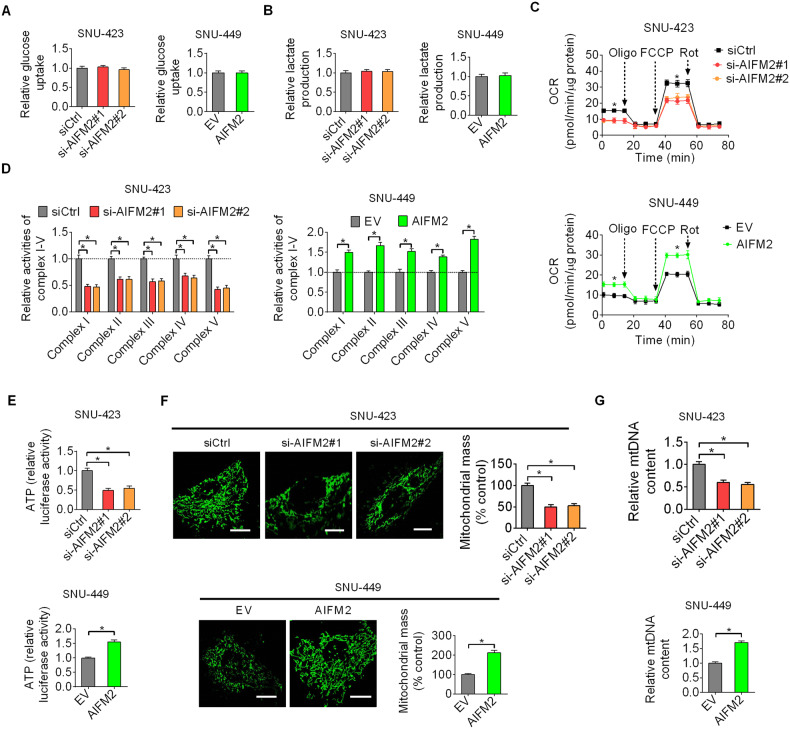
AIFM2-promoted mitochondrial biogenesis and oxidative phosphorylation in HCC cells. **A** and **B** Glucose uptake (**A**) and lactate production (**B**) were determined in AIFM2 knockdown or overexpression HCC cells. **C**–**E** The rate of oxygen consumption (**C**), activities of OXPHOS complexes (**D**) and production of ATP (**E**) were evaluated in AIFM2 knockdown or overexpression HCC cells. (**F**) Confocal microscopy analysis of mitochondrial mass in AIFM2 knockdown or overexpression HCC cells. Scale bars, 5 μm. (**G**) The content of mtDNA was measured by qPCR in AIFM2 knockdown or overexpression HCC cells.

Given that AIFM2 was reported to play a critical role in glucose metabolism regulation, we, therefore, explored the effects of AIFM2 on glucose uptake and lactate production in HCC cells. Unexpectedly, we did not observe any significant changes in glucose uptake and lactate production in AIFM2 knockdown or overexpression HCC cells, as compared with their control cells (Fig. 5A, B). We next determined the effects of AIFM2 on mitochondrial metabolism by evaluating oxygen consumption rate (OCR), OXPHOS complexes activities, and ATP production. The results showed that AIFM2 knockdown markedly suppressed the rate of oxygen consumption, activities of OXPHOS complexes, and production of ATP in SNU-423 and SNU-449 cells, while AIFM2 overexpression significantly increased these mitochondrial oxidative metabolic phenotypes (Fig. 5C–E). In agreement with these mitochondrial respiratory phenotypes, mitochondrial membrane potential was also markedly decreased or increased when AIFM2 was either knocked-down or overexpressed (Fig. S4). Confocal microscopy analysis of mitochondrial morphology showed that AIFM2 knockdown resulted in a significant decrease of mitochondrial mass in SNU-423 cells, while AIFM2 overexpression markedly increased mitochondrial mass in SNU-449 cells (Fig. 5F). In agreement with this, the content of mitochondrial DNA (mtDNA) was also markedly increased or decreased when AIFM2 was either overexpressed or knocked-down in HCC cells (Fig. 5G). The above results indicate that upregulation of AIFM2 increases mitochondrial biogenesis and oxidative phosphorylation in HCC cells.

### AIFM2-promoted mitochondrial biogenesis and oxidative phosphorylation through activation of SIRT1/PGC-1α signaling

**Fig. 6:**
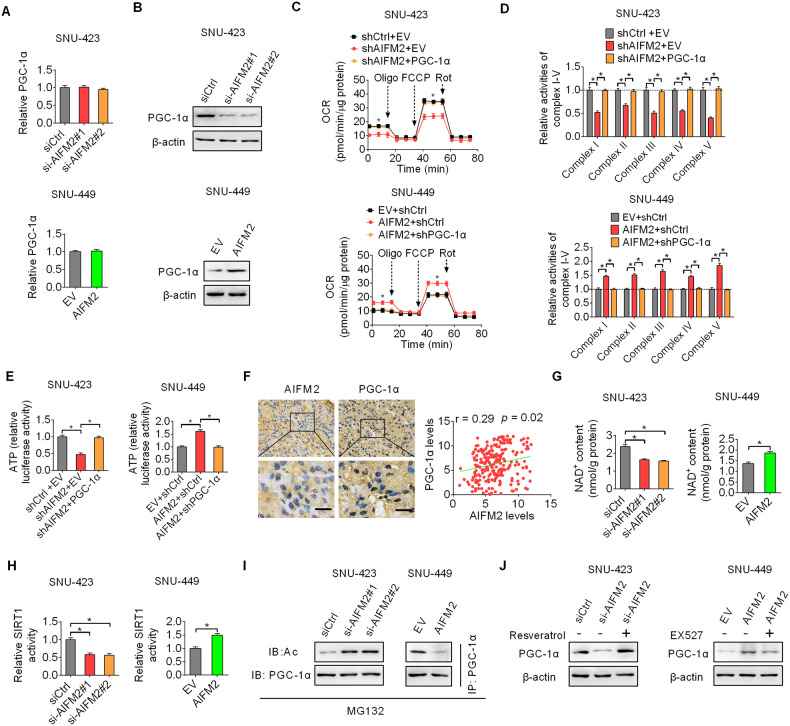
AIFM2-promoted mitochondrial biogenesis and oxidative phosphorylation through activation of SIRT1/PGC-1α signaling. **A** and **B** AIFM2 and PGC-1α expressions were examined by qRT-PCR (**A**) and western blot (**B**) analysis in AIFM2 knockdown or overexpression HCC cells. **C**–**E** The rate of oxygen consumption (**C**), activities of OXPHOS complexes (**D**), and production of ATP (**E**) were evaluated in HCC cells with indicated treatment. (**F**) Correlation between the expressions of AIFM2 and PGC-1α expression at the protein level was analyzed by IHC staining assay in tumor tissues of HCC. Scale bars, 20 μm. **G** and **H** The levels of NAD^+^ (**G**) and activity of SIRT1 (**H**) were determined in AIFM2 knockdown or overexpression HCC cells. **I** Acetylation of PGC-1α was examined by western blot analysis in HCC cells with the treatment of MG132 (a proteasome inhibitor). **J** PGC-1α expression was examined by western blot analysis in HCC cells with the treatment of resveratrol or EX-527.

To gain insight into the molecular mechanistic basis of the promotive effect of AIFM2 on mitochondrial biogenesis and oxidative phosphorylation, we determined the effect of AIFM2 on the expression of PGC-1α (a major regulator of mitochondrial biogenesis). The results showed that PGC-1α expression at the protein level, but not at the mRNA level, was markedly down- or upregulated when AIFM2 is either knocked down or overexpressed in HCC cells (Fig. 6A, B), suggesting that PGC-1α is post-transcriptionally upregulated by AIFM2. We then determined whether PGC-1α was involved in AIFM2-promoted mitochondrial biogenesis and oxidative phosphorylation. The results showed that overexpression of PGC-1α markedly reversed the inhibitory effects of AIFM2 knockdown on the rate of oxygen consumption, activities of OXPHOS complexes, and production of ATP. By contrast, the knockdown of PGC-1α significantly attenuated the promotive effects of AIFM2 overexpression on the rate of oxygen consumption, activities of OXPHOS complexes, and production of ATP in SNU-449 cells (Fig. 6C–E). As expected, a significant positive correlation was found in tumor tissues of HCC between the expressions of AIFM2 and PGC-1α at the protein level, as evaluated by IHC staining assay (Fig. 6F). The above results suggest that AIFM2 promotes mitochondrial biogenesis and oxidative phosphorylation by upregulating PGC-1α expression.

We further determined the molecular mechanism by which AIFM2 upregulated PGC-1α expression in HCC cells. Given that sirtuin 1 (SIRT1), a crucial NAD^+^-dependent protein deacetylase has been demonstrated to upregulate the protein expression level of PGC-1α through deacetylation [11, 12], we, therefore, explored the involvement of SIRT1 in AIFM2-upregulated PGC-1α. The results showed that the level of NAD^+^ and activity of SIRT1 were significantly decreased in SNU-423 cells when AIFM2 was knocked, while increased in SNU-449 cells when AIFM2 was overexpressed (Fig. 6G, H). As expected, the acetylation of PGC-1α was also clearly up- or downregulated, when AIFM2 was knocked-down or overexpressed, in the presence of MG132 (a proteasome inhibitor) (Fig. 6I). Meanwhile, the effect of AIFM2 on the activity of SIRT2, which is another cytoplasmic isoform of SIRT, was also explored. Contrary to our original thought that AIFM2 would affect the activity of SIRT2, its activity was only slightly decreased or increased, but not statistically different, upon AIFM2 silencing or overexpression in SNU-423 and SNU-449 cells (Fig. S5). Moreover, we found that the downregulated PGC-1α expression by AIFM2 knockdown was markedly rescued when SIRT1 was activated by resveratrol (an activator of SIRT1) treatment, while the upregulated PGC-1α expression by forced AIFM2 expression was markedly attenuated when SIRT1 was activated by suppressed by EX-527 (an inhibitor of SIRT1) treatment (Fig. 6J). Collectively, these findings suggest that AIFM2 promotes mitochondrial biogenesis and oxidative phosphorylation in HCC cells through activation of SIRT1/PGC-1α signaling.

### AIFM2-promoted HCC metastasis through increasing PGC-1α-regulated mitochondrial biogenesis

**Fig. 7:**
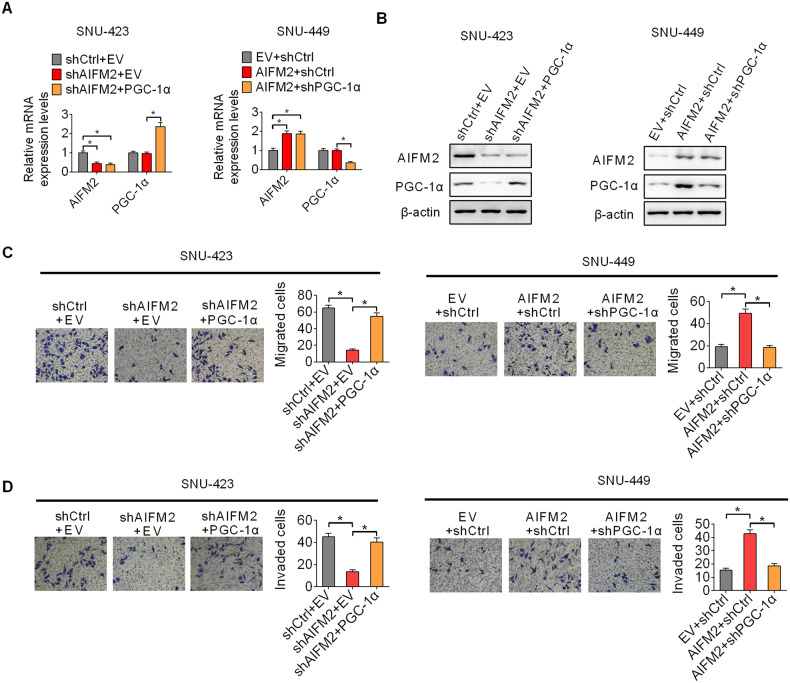
AIFM2-promoted HCC metastasis through increasing PGC-1α-regulated mitochondrial biogenesis. **A** and **B** Overexpression or knockdown of PGC-1α was examined by qRT-PCR (**A**) and western blot (**B**) analysis in HCC cells with indicated treatment. **C** and **D** Transwell migration (**C**) and invasion (**D**) assays in HCC cells with indicated treatment.

Previous studies have shown that PGC-1α-regulated mitochondrial biogenesis plays a crucial role in the promotion of metastasis in several types of human cancers [13–16]. To explore the involvement of PGC-1α-regulated mitochondrial biogenesis in AIFM2-promoted metastasis of HCC, we suppressed mitochondrial biogenesis by knocking of PGC-1α (Fig. 7A, B). The results indicated that activation of mitochondrial biogenesis by forced PGC-1α expression markedly reversed the migration and invasion of SNU-423 and HLF cells suppressed by AIFM2 knockdown (Fig. 7C, D). By contrast, suppression of mitochondrial biogenesis by PGC-1α knocking-down markedly attenuated the migration and invasion of SNU-449 and Hep3B cells promoted by forced AIFM2 expression. These results suggest that AIFM2 promotes HCC metastasis through enhancing PGC-1α-regulated mitochondrial biogenesis.

## Discussion

AIFM2 has been identified as a crucial NADH oxidase involved in the regulation of cytosolic NAD^+^ [4]. Previous studies have revealed that the expression of AIFM2 is low in various organs but high in brown adipose tissue (BAT) [17]. However, the expression of AIFM2 in tumor tissues remains largely unknown. In this study, we for the first time demonstrate that AIFM2 is frequently upregulated in HCC tissue samples and cell lines. AIFM2 expression is positively associated with the clinicopathological feature of tumor metastasis in patients with HCC. Additionally, High expression of AIFM2 is significantly associated with poor patient survival. These findings indicate AIFM2 as a potential prognostic marker in HCC.

Significant upregulation of AIFM2 in tumor tissues of HCC promoted us to investigate the potential oncogenic functions of AIFM2 in HCC progression. In vitro cell proliferation, migration, and invasion assays demonstrated that knockdown of AIFM2 significantly impaired, while forced expression of AIFM2 markedly enhanced the migration and invasion of HCC cells. However, no notable changes in short- and long-term cell proliferation, apoptosis, and cell cycle progression were observed when AIFM2 was either knocked down or overexpressed. Consistent with our findings in HCC cells, Aifm2 was also reported to have no effect on apoptosis in BAT cells [4]. In addition, a recent study also has suggested that AIFM2 facilitated the metastasis of HER-2-positive breast cancer cells [18]. In line with the in vitro findings, in vivo, lung metastasis nude mouse models also demonstrated that AIFM2 upregulation significantly increased the metastatic tumor nodules formed in the lungs. These findings collectively suggest that AIFM2 overexpression promoted HCC metastasis both in vitro and in vivo.

Upregulation of AIFM2 in HCC at both mRNA and protein levels suggests that its upregulation may occur at the pre-translational level. It has been well-established that cancer cells are characterized by aberrant DNA methylation [19]. We found a negative correlation between the DNA methylation and mRNA expression levels of AIFM2 in HCC, suggesting that the hypomethylation of the AIFM2 promoter may contribute to the upregulation of AIFM2 in HCC. Additionally, microRNAs (miRNAs) are small non-protein coding RNAs that participate in the regulation of almost every biological process. Deregulation in miRNAs expression has been demonstrated in various human cancers by acting as oncogenes or tumor suppressors. Studies in several types of cancers, such as colorectal [20–22], ovarian [23], non-small-cell lung [24], pancreatic [25], nasopharyngeal [26], and glioma [27] cancers, have revealed markedly downregulated levels of miR-150-5p, which plays important suppressive functions in tumor growth and metastasis. In HCC, miR-150-5p was also reported to be downregulated and function as a suppressor of hepatoma cell migration and invasion [28]. In agreement with this, we also found a significant decrease of miR-150-5p in HCC tumor tissues as compared with paired non-tumor tissues. In addition, we demonstrate that the upregulation of AIFM2 in HCC is mainly caused by decreased miR-150-5p. Further, we found that transfection with miR-150-5p greatly attenuated AIFM2-promoted HCC cell migration and invasion. However, given that gene expression regulation occurs at multiple levels, we cannot exclude the possibility that other factors may also contribute to the upregulation of AIFM2 in HCC, which still needs more investigation.

Metabolism reprogramming has been well-known as a hallmark of cancer [29]. Given that AIFM2 was reported to regulate glucose metabolism in brown adipose tissue cells [4], we therefore explored whether AIFM2 also participates in glucose metabolism regulation in HCC cells. Unexpectedly, no significant effects on glucose uptake and lactate production were observed when AIFM2 was knocked down or overexpressed, whereas oxygen consumption rate, OXPHOS complexes activities, and ATP production were positively regulated by AIFM2 in HCC cells, indicating that AIFM2 promotes mitochondrial oxidative metabolism in HCC cells. In addition, we found that AIFM2-promoted mitochondrial oxidative metabolism by upregulating PGC-1α-mediated mitochondrial biogenesis.

SIRT1 is an NAD^+^-dependent deacetylase that has been demonstrated to upregulate the protein expression level of PGC-1α through deacetylation [12, 30]. Consistently, we also found in HCC cells that AIFM2 upregulation increased the level of NAD^+^ and SIRT1 activation, which subsequently upregulated the protein expression level of PGC-1α by deacetylating it. Furthermore, we demonstrated that AIFM2-promoted HCC metastasis through increasing PGC-1α-regulated mitochondrial biogenesis. In line with our findings, several previous studies also have shown that PGC-1α-regulated mitochondrial biogenesis plays crucial roles in the promotion of metastasis in several types of human cancers [13–16].

**Fig. 8:**
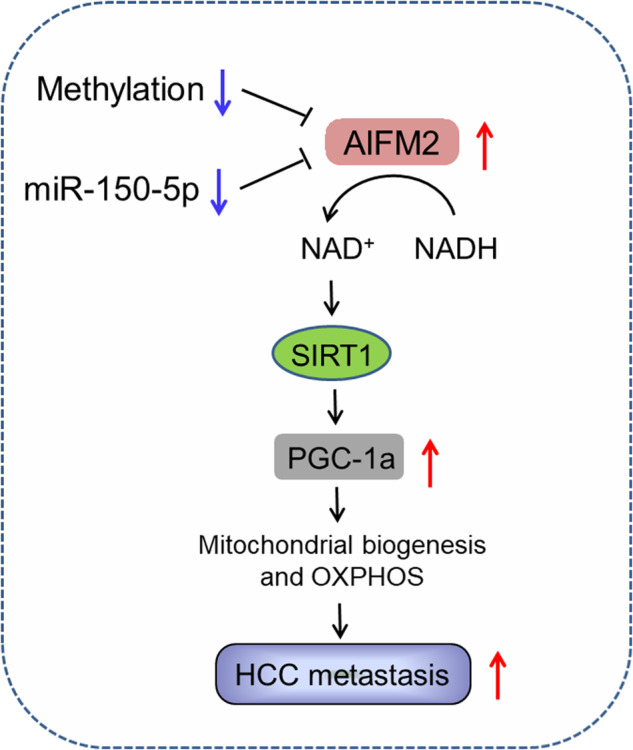
Schematic figure showing the crucial roles of AIFM2 in the promotion of HCC metastasis. AIFM2 is frequently overexpressed in HCC, which is probably caused by DNA hypomethylation and downregulation of miR-150-5p. AIFM2 plays a crucial role in the promotion of tumor metastasis by increasing mitochondrial biogenesis and oxidative phosphorylation through activation of NAD+/SIRT1/PGC-1α signaling.

In summary, we demonstrate that AIFM2 is frequently overexpressed in HCC and plays a crucial role in the promotion of tumor metastasis by increasing mitochondrial biogenesis and oxidative phosphorylation through activation of NAD^+^/SIRT1/PGC-1α signaling (Fig. 8). These findings suggest AIFM2 as a potential prognostic marker and therapeutic target in HCC.

### Supplementary information

Supplementary figures and tables

Original Data File

## Data Availability

The data that support the findings of this study are available from the corresponding author upon reasonable request.
