# Supplementary material for: AIFM2 promotes hepatocellular carcinoma metastasis by enhancing mitochondrial biogenesis through activation of SIRT1/PGC-1α signaling
Source: Oncogenesis. 2023 Sep 21;12(1):46. doi: 10.1038/s41389-023-00491-1 (PMC10514190; doi:10.1038/s41389-023-00491-1)
Supplement: Supplementary file 1 — Supplementary figures and tables [file 41389_2023_491_MOESM1_ESM.docx]

**Supplemental information**

**AIFM2 promotes hepatocellular carcinoma metastasis by enhancing mitochondrial biogenesis through activation of SIRT1/PGC-1α signaling**

**Supplemental figures**

**Figure S1.** The expression patterns of AFIM2 were analyzed in 26 types of human cancers using the online database Sangerbox (<http://vip.sangerbox.com/>).

**
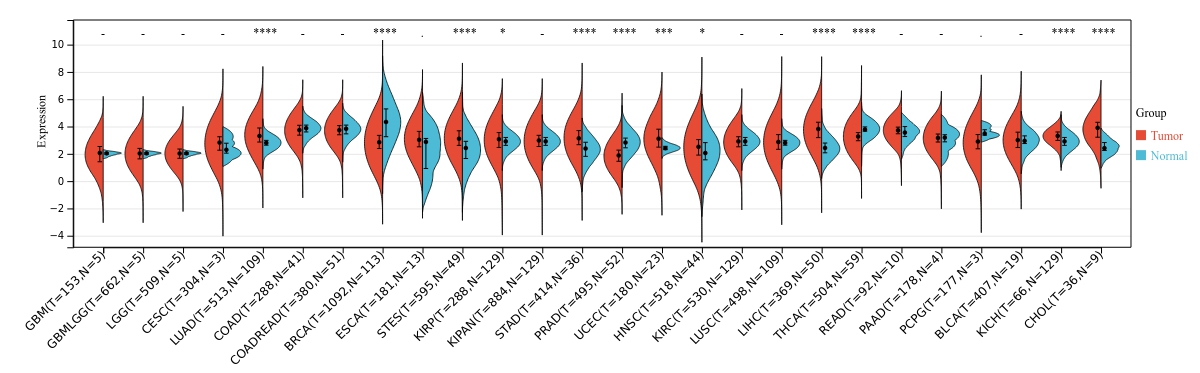
**

**Figure S2.** **(A and B)** Stable knockdown of AIFM2 in SNU-423 cells was confirmed by qRT-PCR (A) and western blot (B) assays. (C and D) Stable overexpression of AIFM2 in SNU-449 cells was confirmed by qRT-PCR (A) and western blot (B) assays.





**Figure S3.** Top three predicted miRNAs targeting AIFM2 based on the online mirDIP platform.


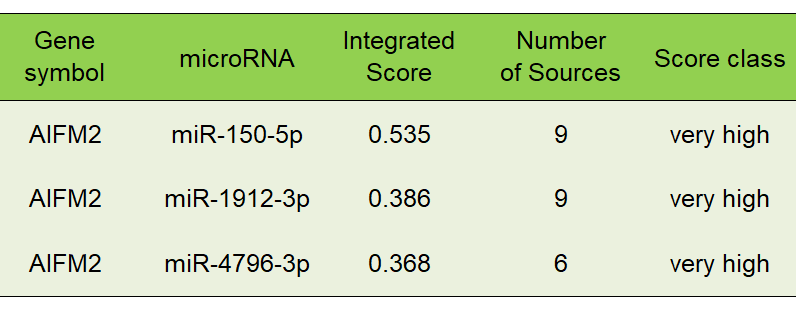


**Figure S4.** The effect of AIFM2 on mitochondria membrane potential was evaluated by JC-1 staining in SNU-423 and SNU-449 cells with AIFM2 silencing or overexpression. Scale bars, 2 μm.

**
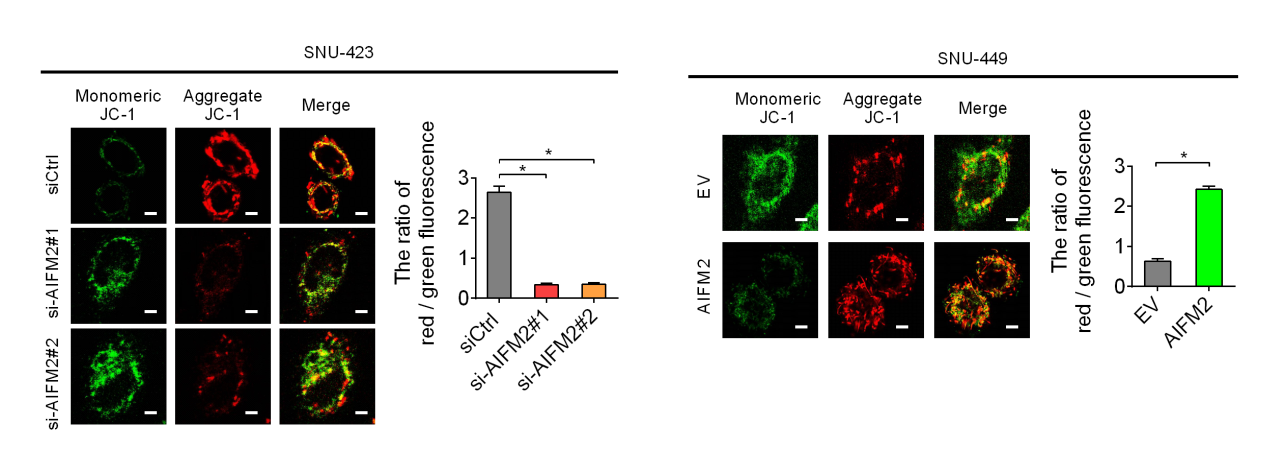
**

**Figure S5.** The effect of AIFM2 on the activity of SIRT2 was explored in SNU-423 and SNU-449 cells with AIFM2 silencing or overexpression.


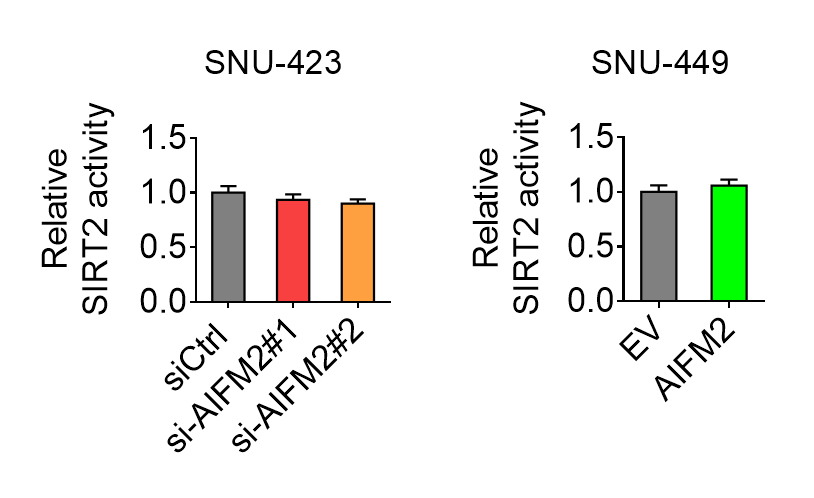


**Supplemental Tables**

**Table S1.** Sequence of primers used for qRT-PCR analysis

| *AIFM2* | forward primer | AGACAGGGTTCGCCAAAAAGA |
| --- | --- | --- |
|  | reverse primer | CAGGTCTATCCCCACTACTAGC |
| *PGC-1a* | forward primer | TCTGAGTCTGTATGGAGTGACAT |
|  | reverse primer | CCAAGTCGTTCACATCTAGTTCA |
| *miR-150-5p* | forward primer | CATGGCCCTGTCTCCCAAC |
|  | reverse primer | GGCCTGTACCAGGGTCTGA |
| *U6* | forward primer | CTCGCTTCGGCAGCACA |
|  | reverse primer | AACGCTTCACGAATTTGCGT |
| *AIFM2*  *unmethylated (U)* | forward primer | GTTATTTTTTGTATGTAGATGTTGA |
|  | reverse primer | TCTCAACCCACTTTAATAACTCAAA |
| *AIFM2*  *methylated (M)* | forward primer | TGGTTATTTTTTGTACGTAGATGTC |
|  | reverse primer | TCAACCCACTTTAATAACTCGAA |
| *HGB* | forward primer | GTGCACCTGACTCCTGAGGAGA |
|  | reverse primer | CCTTGATACCAACCTGCCCAG |
| *ND1* | forward primer | CCCTAAAACCCGCCACATCT |
|  | reverse primer | GAGCGATGGTGAGAGCTAAGGT |
| *β-actin* | forward primer | ATCAAGATCATTGCTCCTCCTGAG |
|  | reverse primer | CTGCTTGCTGATCCACATCTG |

**Table S2.** Primary antibodies used for western bloting and IHC staining assays.

| **Antibody** | **Company (Cat. No.)** | **Working dilutions** |
| --- | --- | --- |
| AIFM2 | Proteintech (20886-1-AP) | WB: 1/1000; IHC:1/250 |
| PGC-1a | Proteintech (66369-1-Ig) | WB: 1/1000; IHC: 1/100 |
| AC-lys | Cell signaling (#9441) | WB: 1/500 |
| β-actin | Proteintech (20536-1-AP) | WB: 1/1000 |

**Table S 3.** **Correlation between AIFM2 expression and clinicopathologic features of 213 HCC patients.**

| Variables | | No. of cases (%) | | | AIFM2 expression | | | | | *P* value | |
| --- | --- | --- | --- | --- | --- | --- | --- | --- | --- | --- | --- |
|  |  |  |  |  | Low | | | High | |  |  |
| All | | 213 (100%) | | | 106 | | | 107 | |  | |
| Age | |  | | |  | | |  | |  | |
| <=55 | | 87 (40.8%) | | | 41 | | | 46 | | 0.578 | |
| >55 | | 126 (59.2%) | | | 65 | | | 61 | |  |  |
| Gender | |  | | |  | | |  | |  | |
| Female | | 35 (16.4%) | | | 15 | | | 20 | | 0.460 | |
| Male | | 178 (83.6%) | | | 91 | | | 87 | |  |  |
| HBV | |  | | |  | | |  | |  | |
| Negative | | 27 (12.7%) | | | 12 | | | 15 | | 0.681 | |
| Positive | | 186 (87.3%) | | | 94 | | | 92 | |  |  |
| AFP (ug/ml) | |  | | |  | | |  | |  | |
| <=200 | | 116 (54.5%) | | | 61 | | | 55 | | 0.410 | |
| >200 | | 97 (45.5%) | | | 45 | | | 52 | |  |  |
| Maximum diameter of lesion | |  | | |  | | |  | |  | |
| <=5 | | 110 (46.9%) | | | 61 | | | 49 | | 0.100 | |
| >5 | | 103 (53.1%) | | | 45 | | | 58 | |  |  |
| PVTT (portal vein tumor thrombosis) | |  | | |  | | |  | |  | |
| No | | 174 (81.7%) | | | | 95 | | 79 | | **0.004** | |
| Yes | | 39 (18.3%) | | | | 11 | | 28 | |  |  |
| TNM stage | | | |  | |  | |  | |  | |
| I+ II | | | | 169 (79.3%) | | 87 | | 82 | | 0.398 | |
| III+ IV | | | | 44 (20.7%) | | 19 | | 25 | |  |  |
| Differentiation grade | | | |  | |  | |  | |  | |
| I+ II | | | | 65 (30.5%) | | 34 | | 31 | | 0.657 | |
| III | | | | 148 (69.5%) | | 72 | | 76 | |  |  |
