# Supplementary material for: AIFM2 promotes hepatocellular carcinoma metastasis by enhancing mitochondrial biogenesis through activation of SIRT1/PGC-1α signaling
Source: Oncogenesis. 2023 Sep 21;12(1):46. doi: 10.1038/s41389-023-00491-1 (PMC10514190; doi:10.1038/s41389-023-00491-1)

Fig 1I

AIFM2

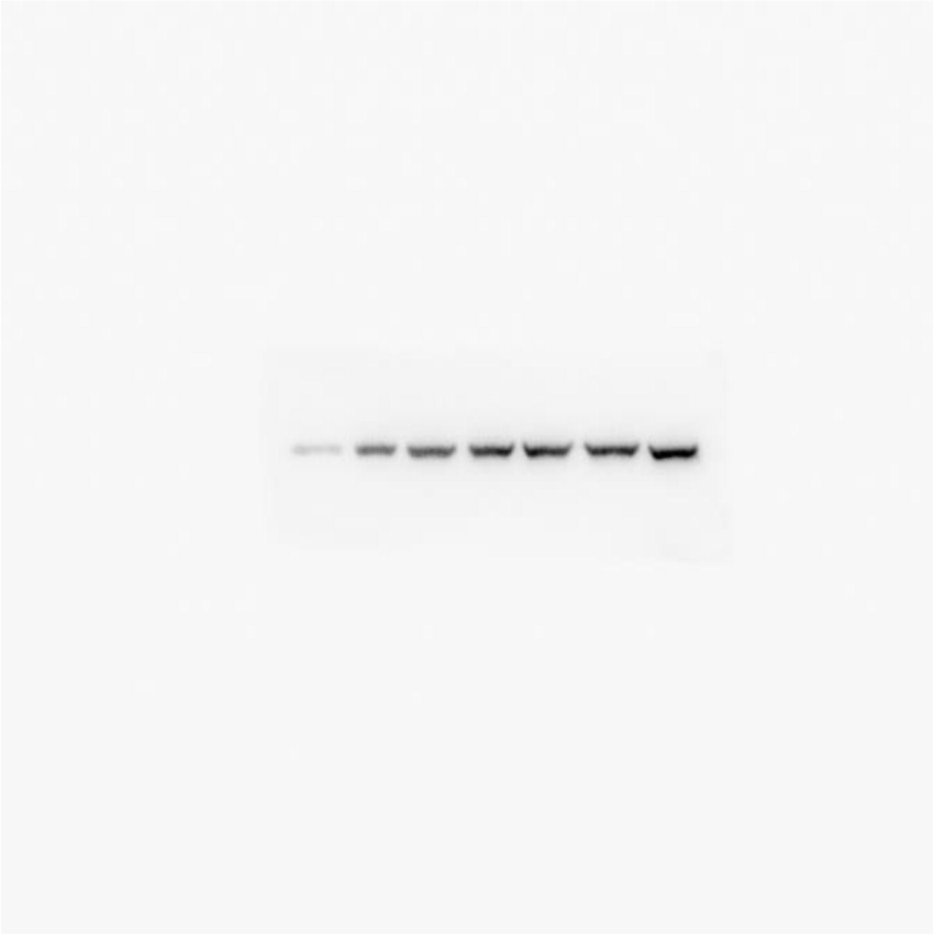

Fig 1I

β-actin

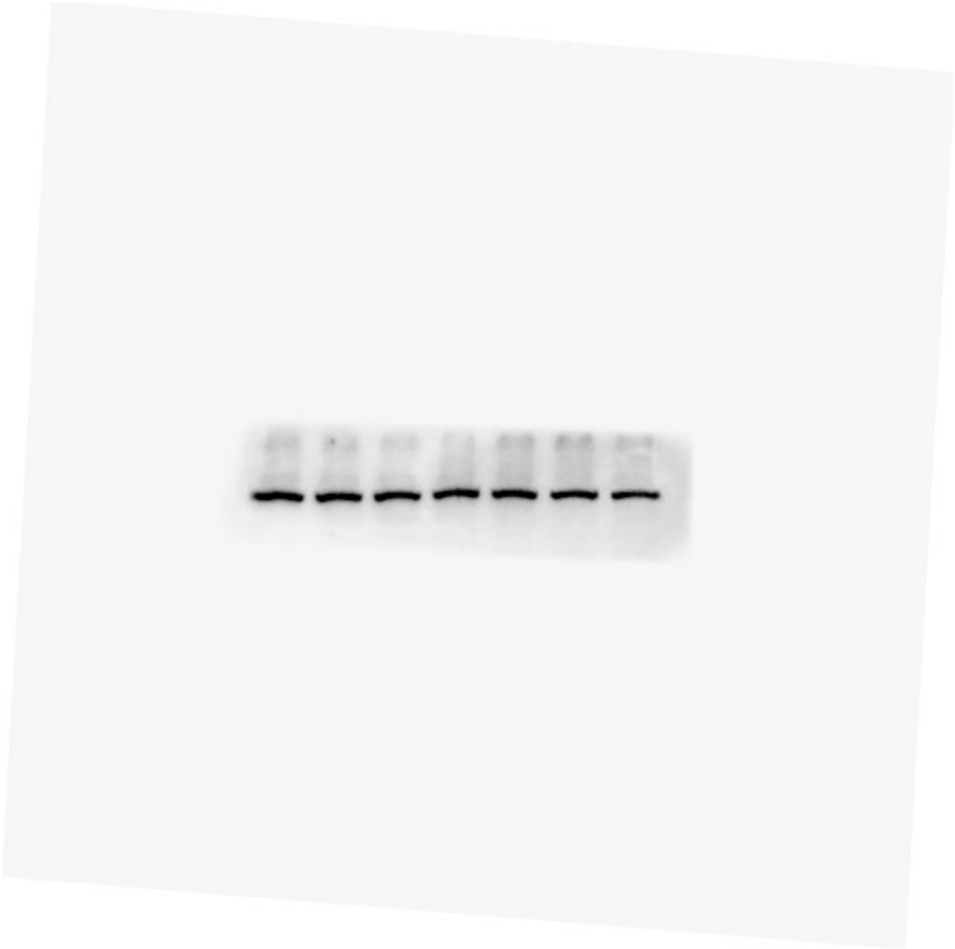

Fig 2B

AIFM2 (SNU-423)

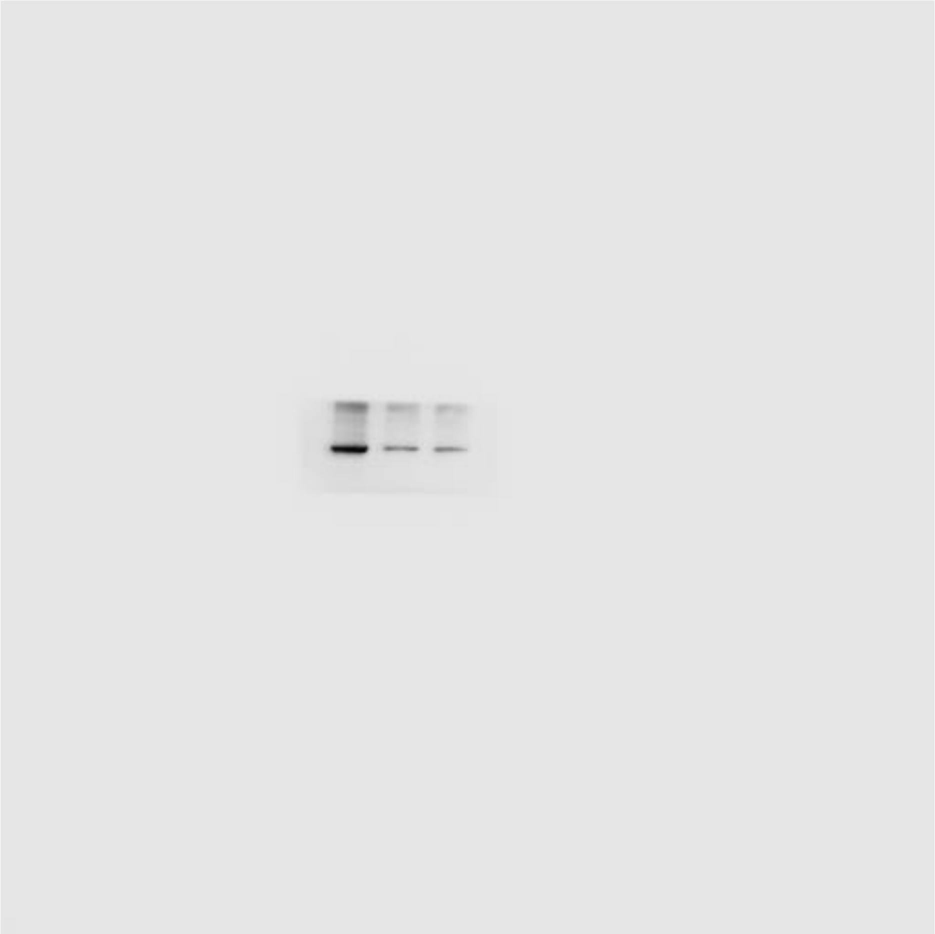

Fig 2B

β-actin (SNU-423)

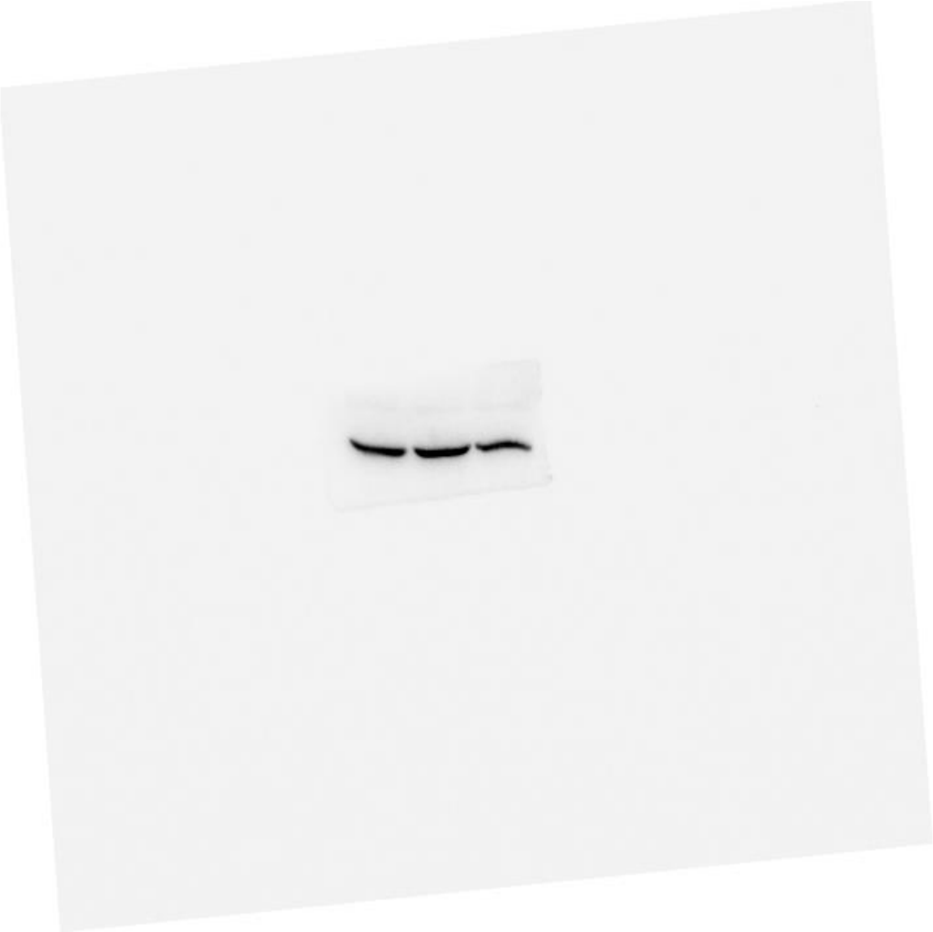

Fig 2B

AIFM2 (HLF)

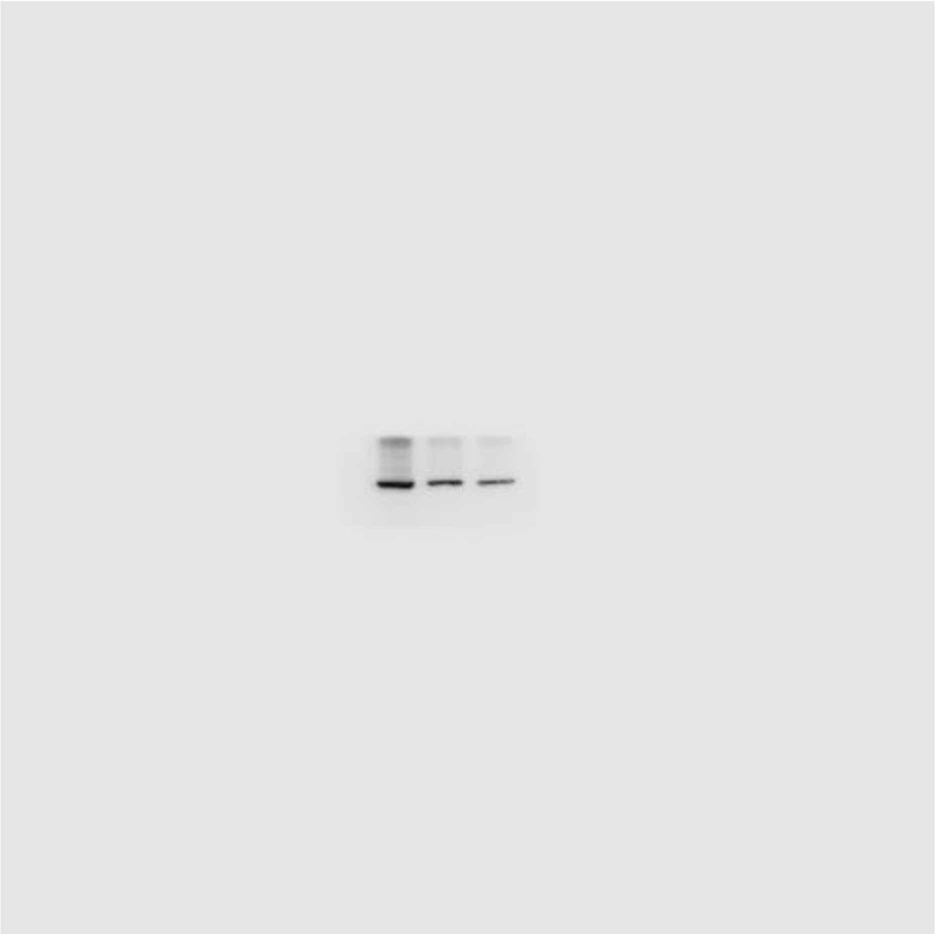

Fig 2B

β-actin (HLF)

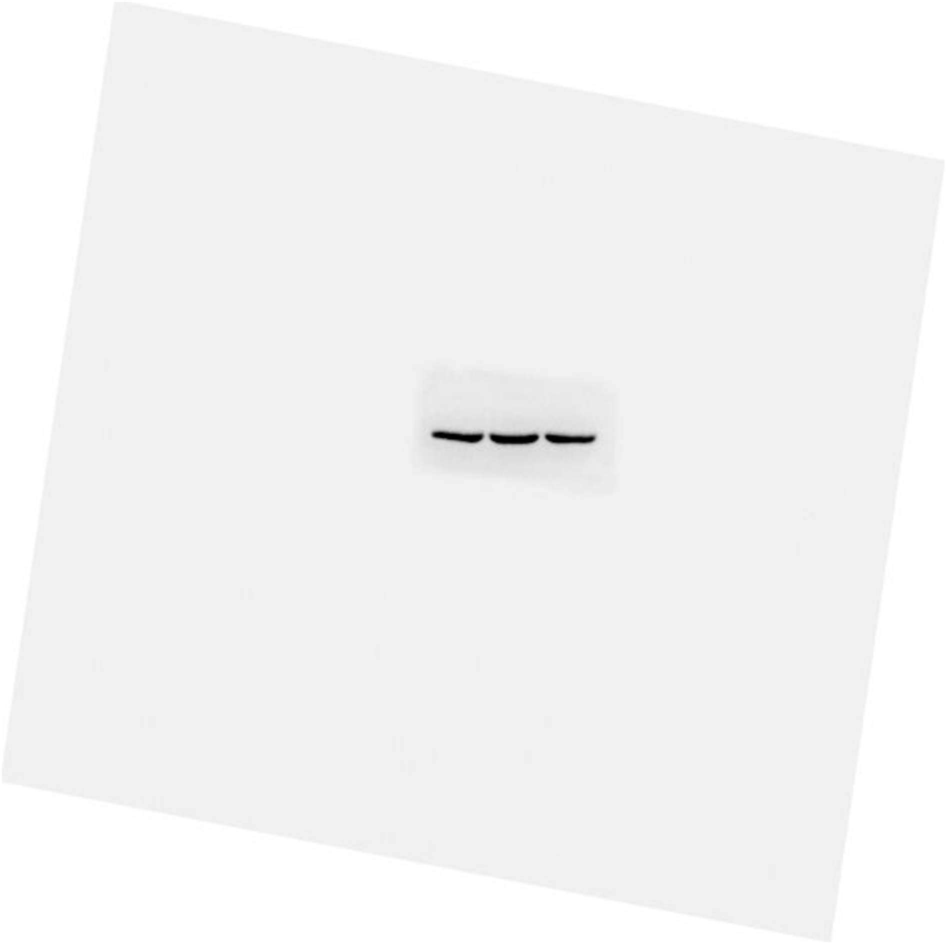

Fig 3B

AIFM2 (SNU-449)

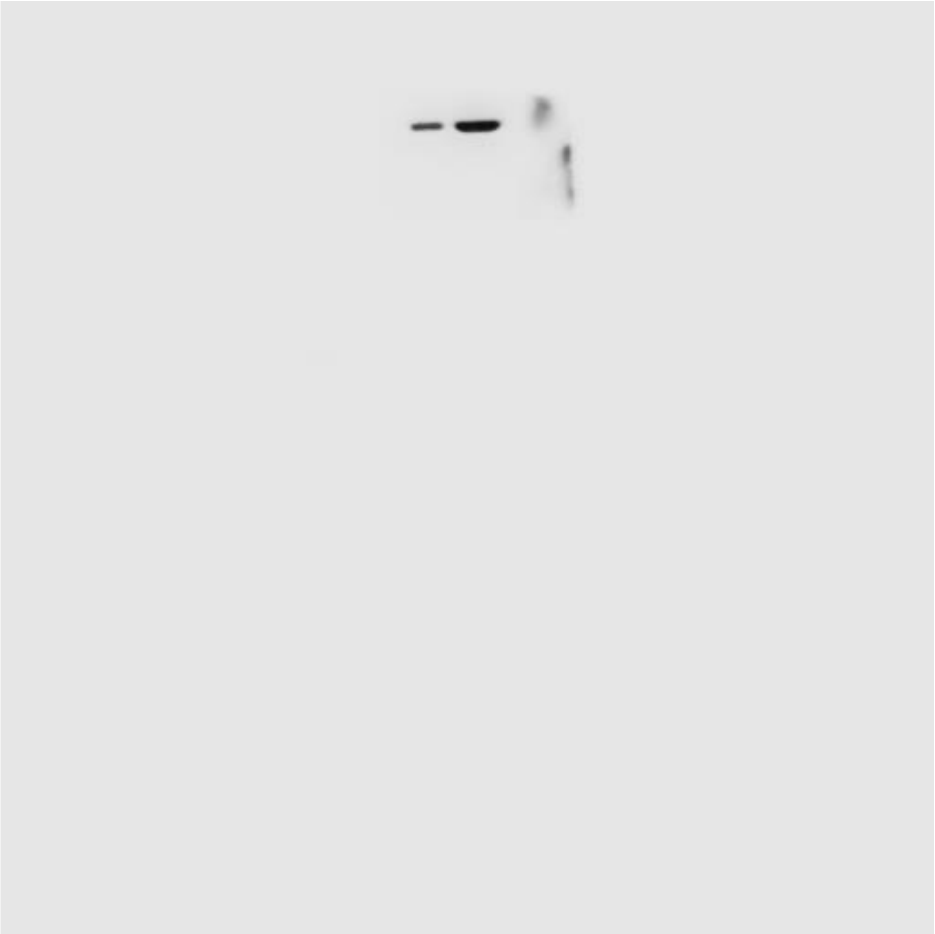

Fig 3B

β-actin (SNU-449)

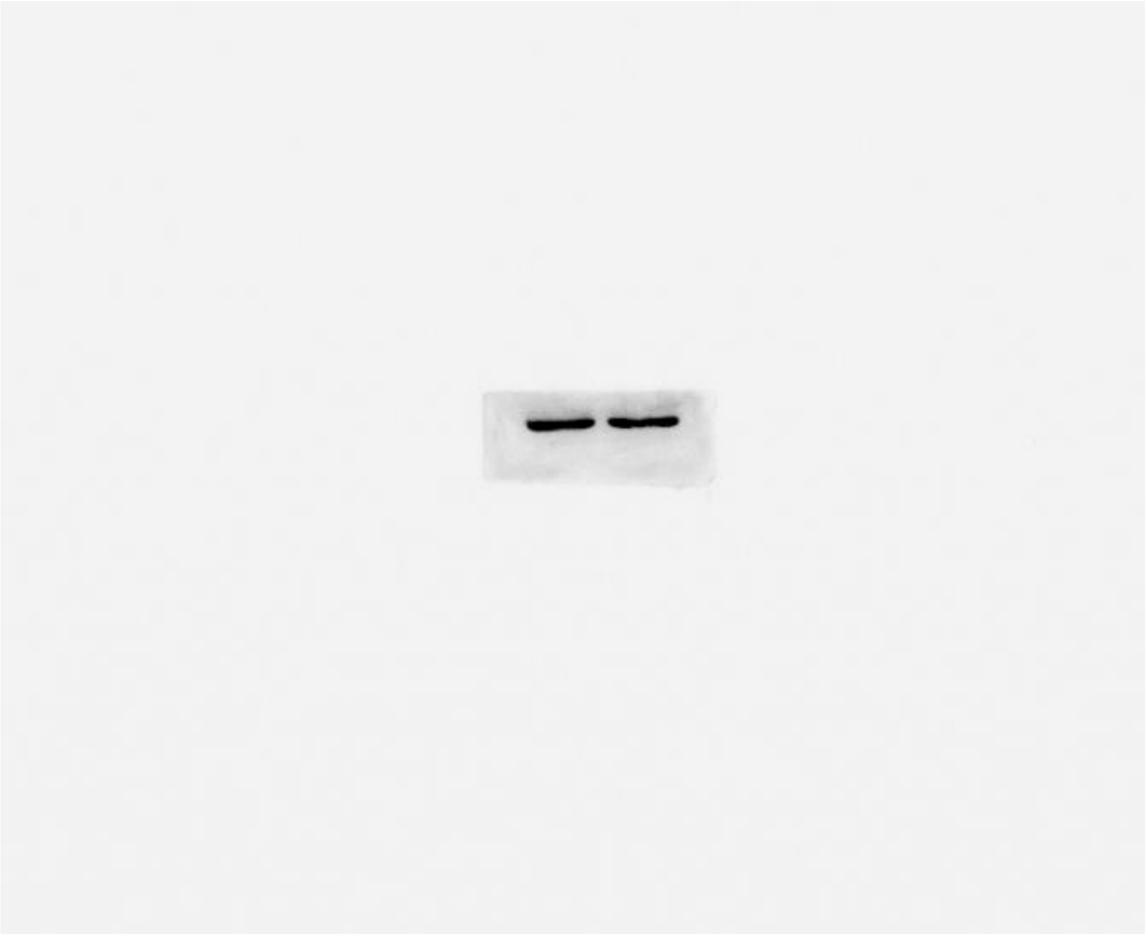

Fig 3B

AIFM2 (Hep3B)

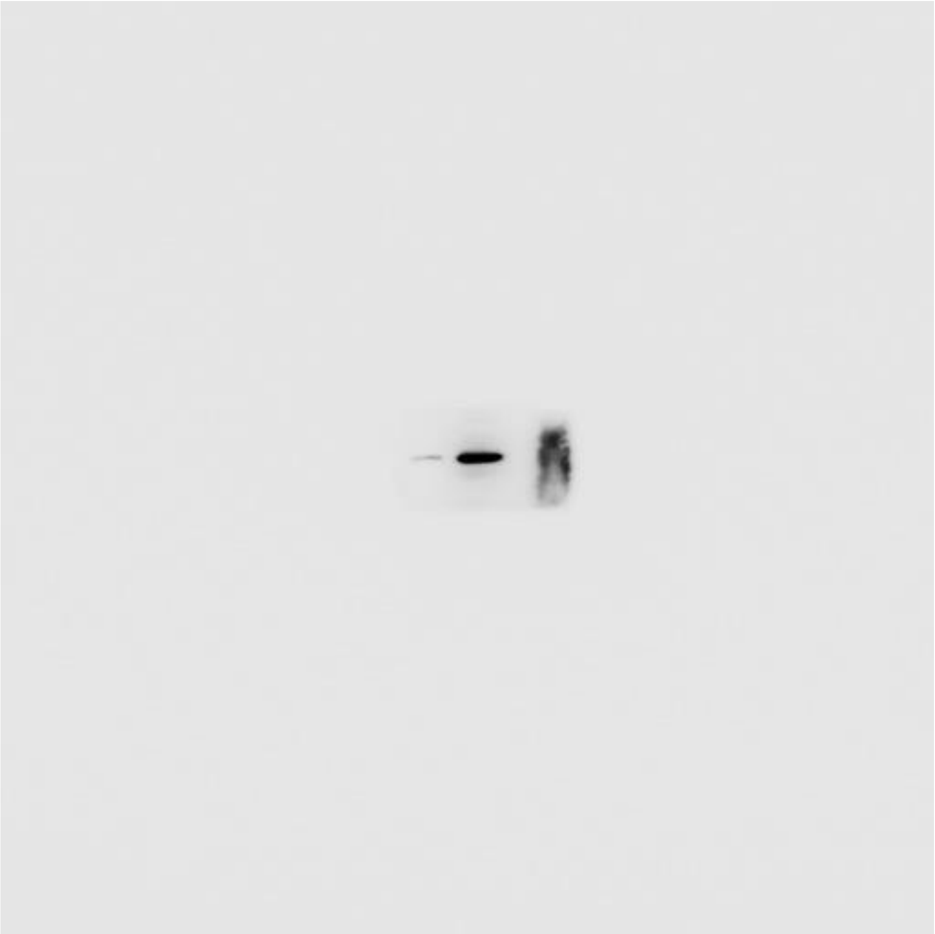

Fig 3B

β-actin (Hep3B)

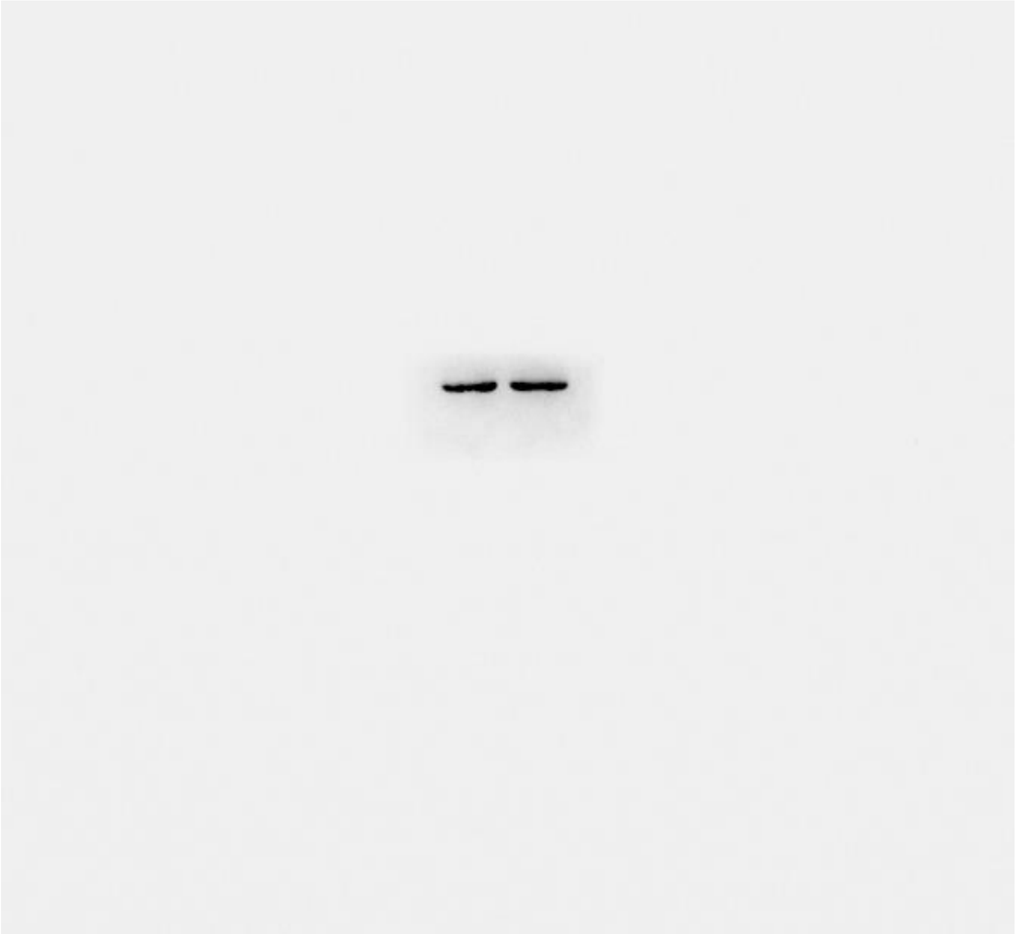

Fig 4E

AIFM2 (SNU-423)

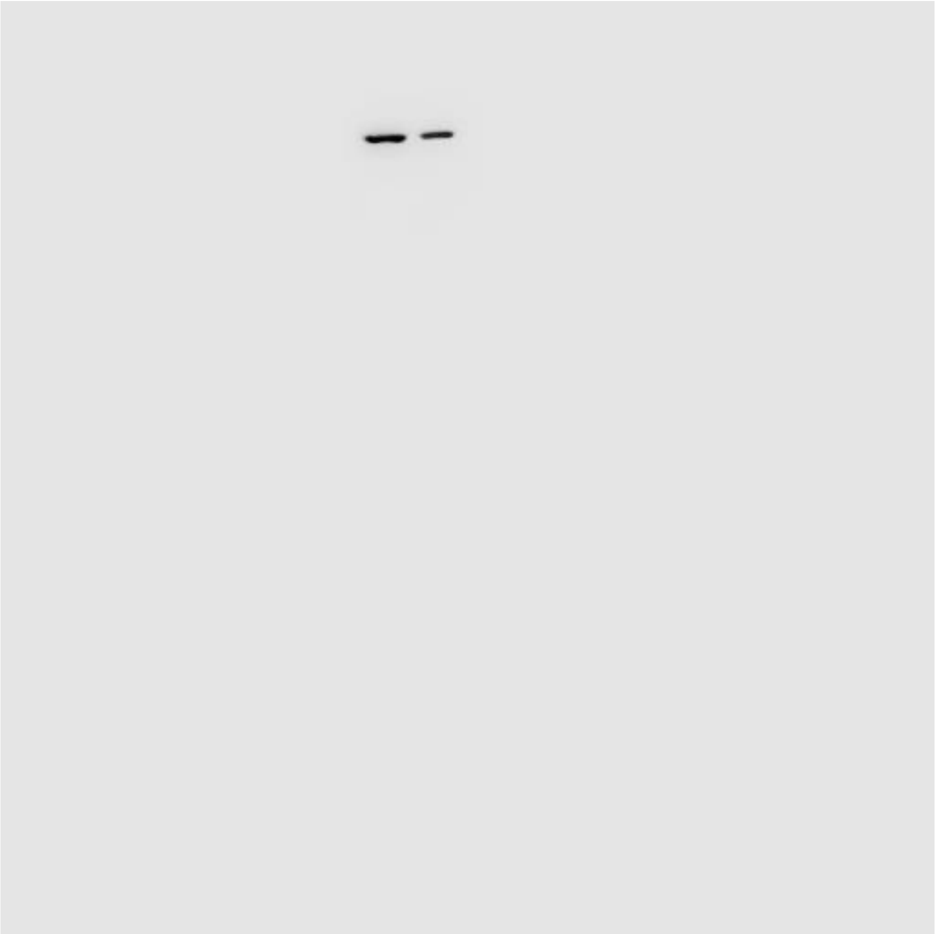

Fig 4E

β-actin (SNU-423)

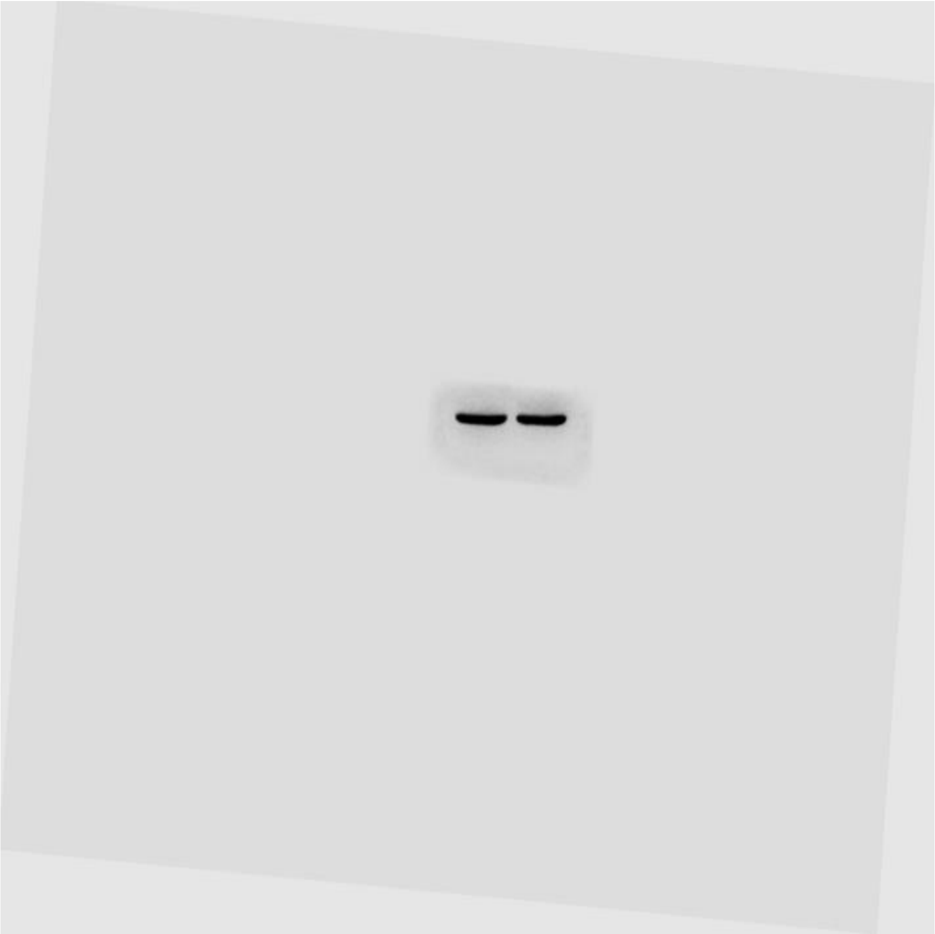

Fig 4E

AIFM2 (HLF)

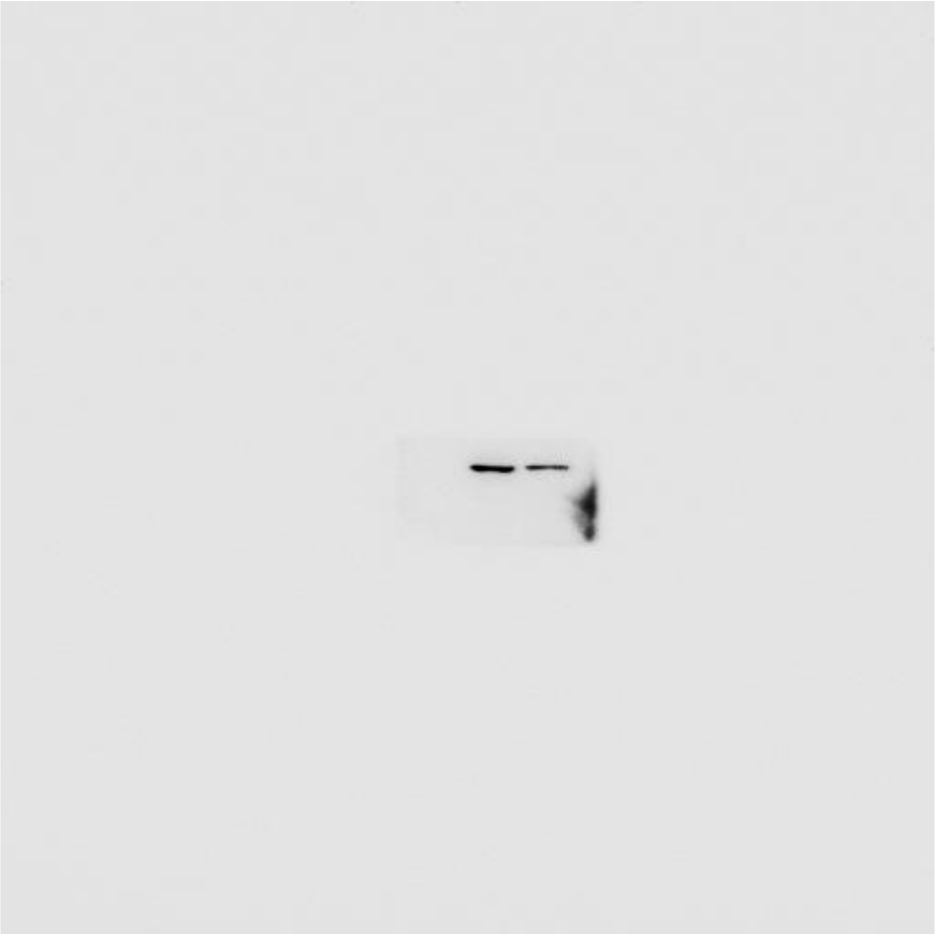

Fig 4E

β-actin (HLF)

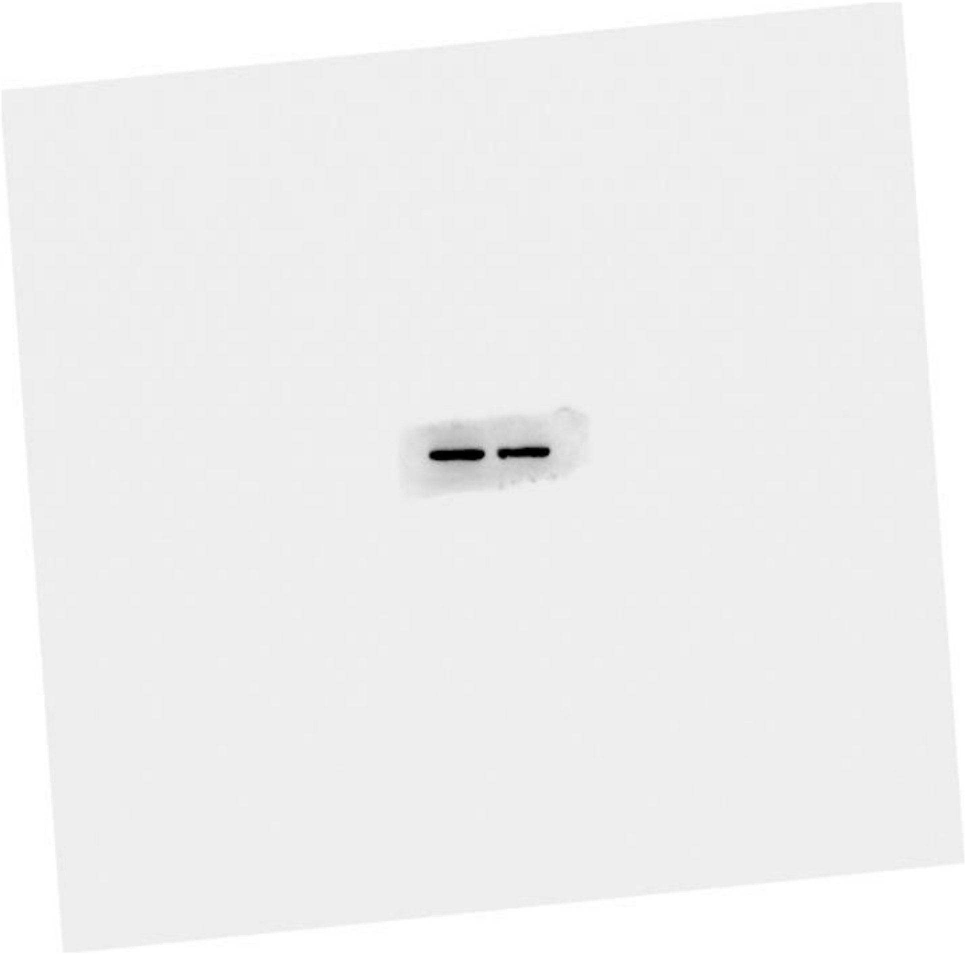

Fig 6B

PGC-1α (SNU-423)

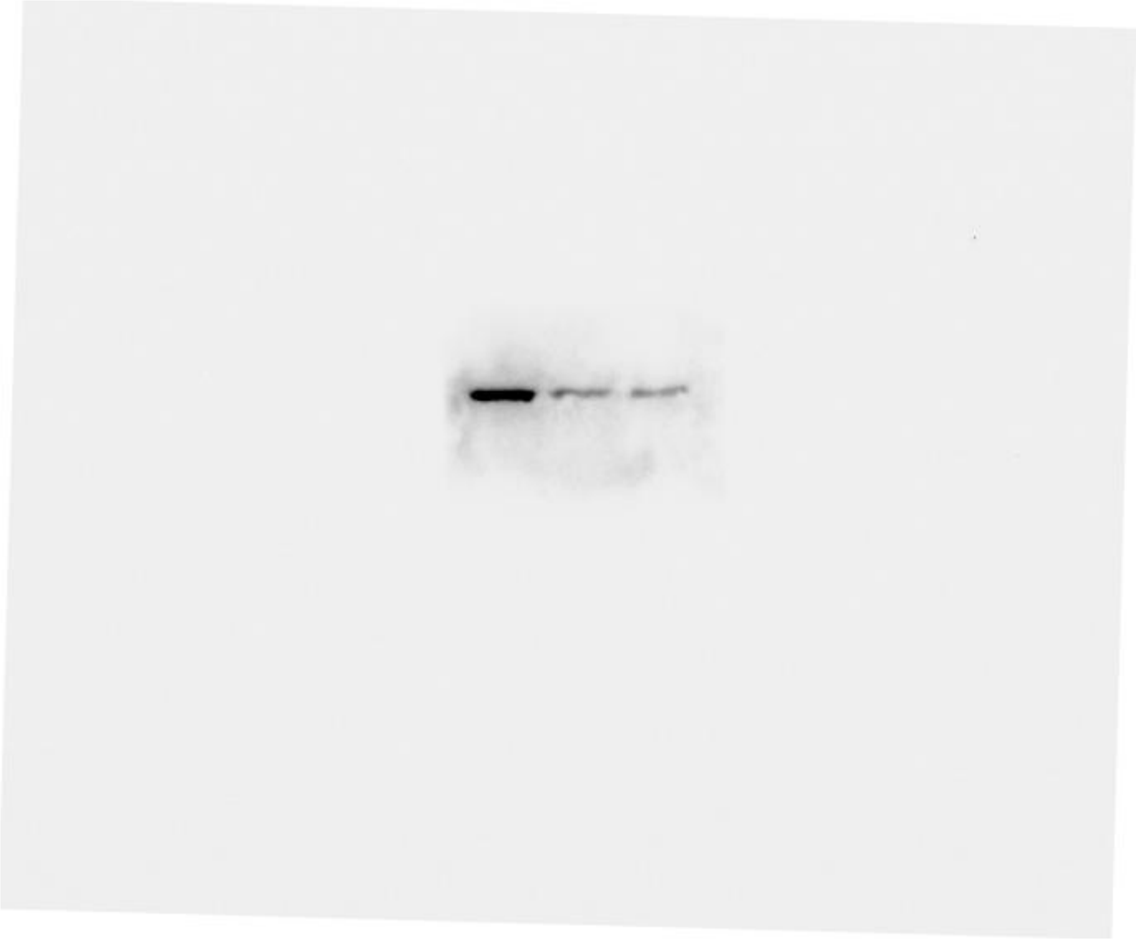

Fig 6B

β-actin (SNU-423)

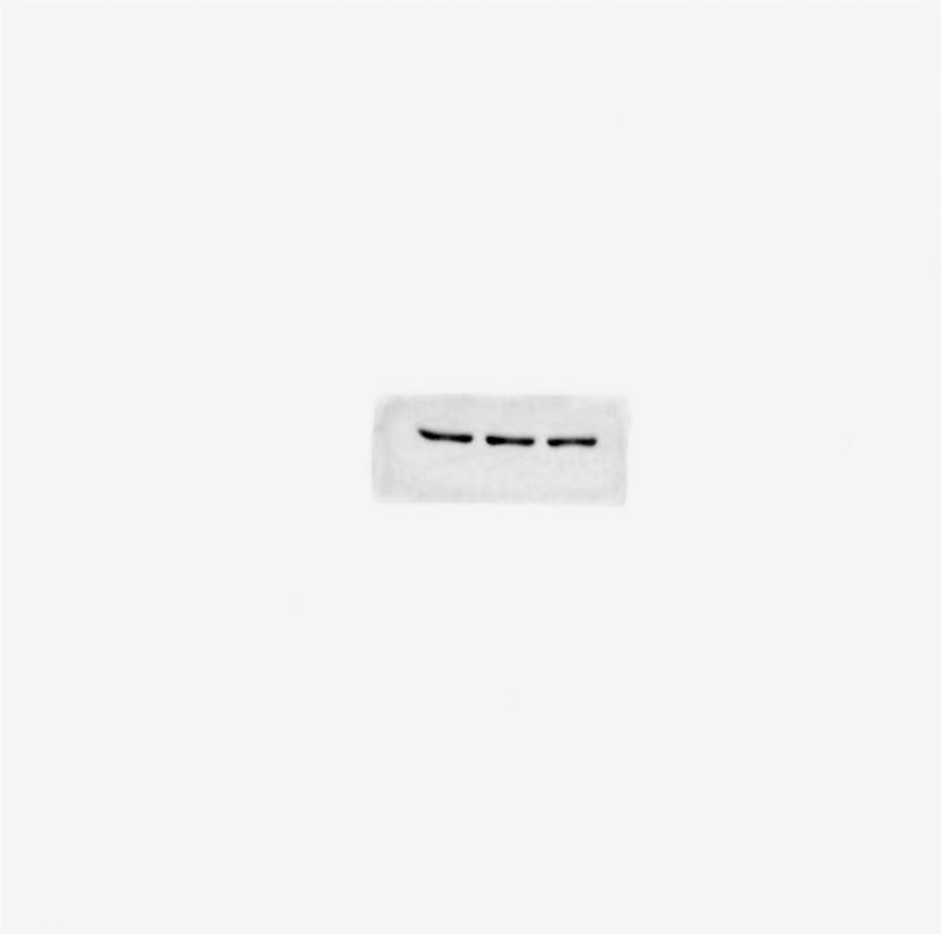

Fig 6B

PGC-1α (SNU-449)

Fig 6B

β-actin (SNU-449)

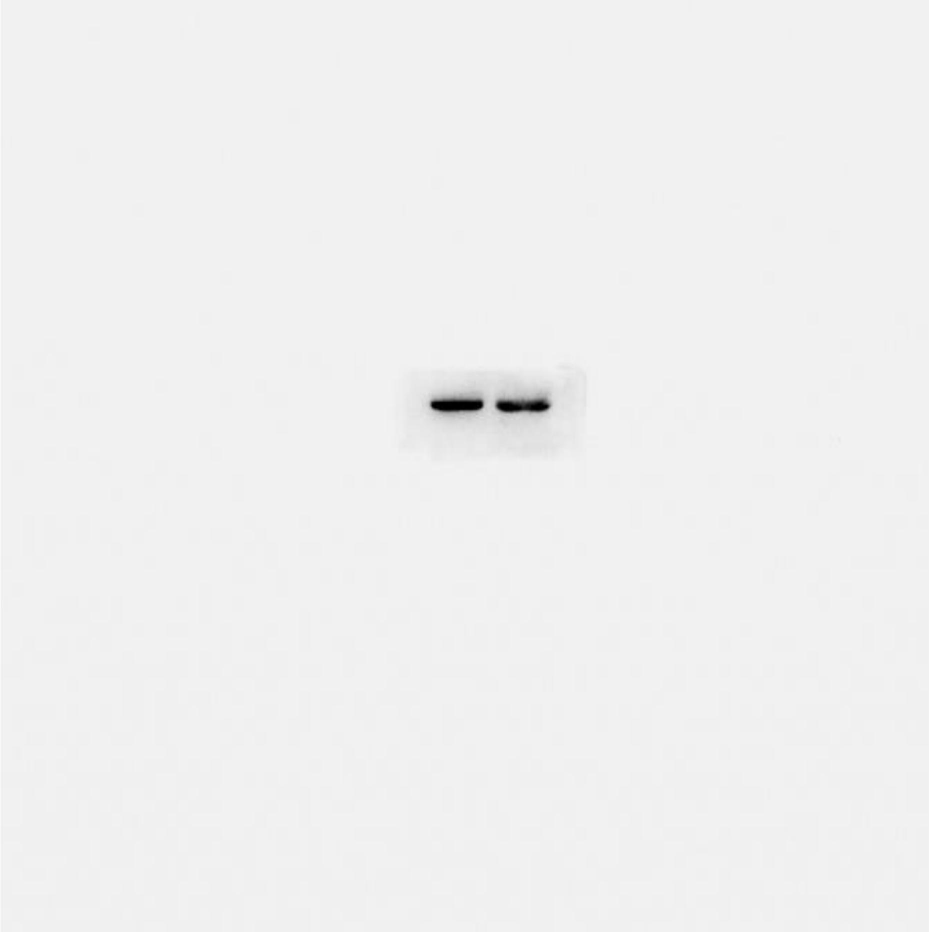

Fig 6I

IB: AC (SNU-423)

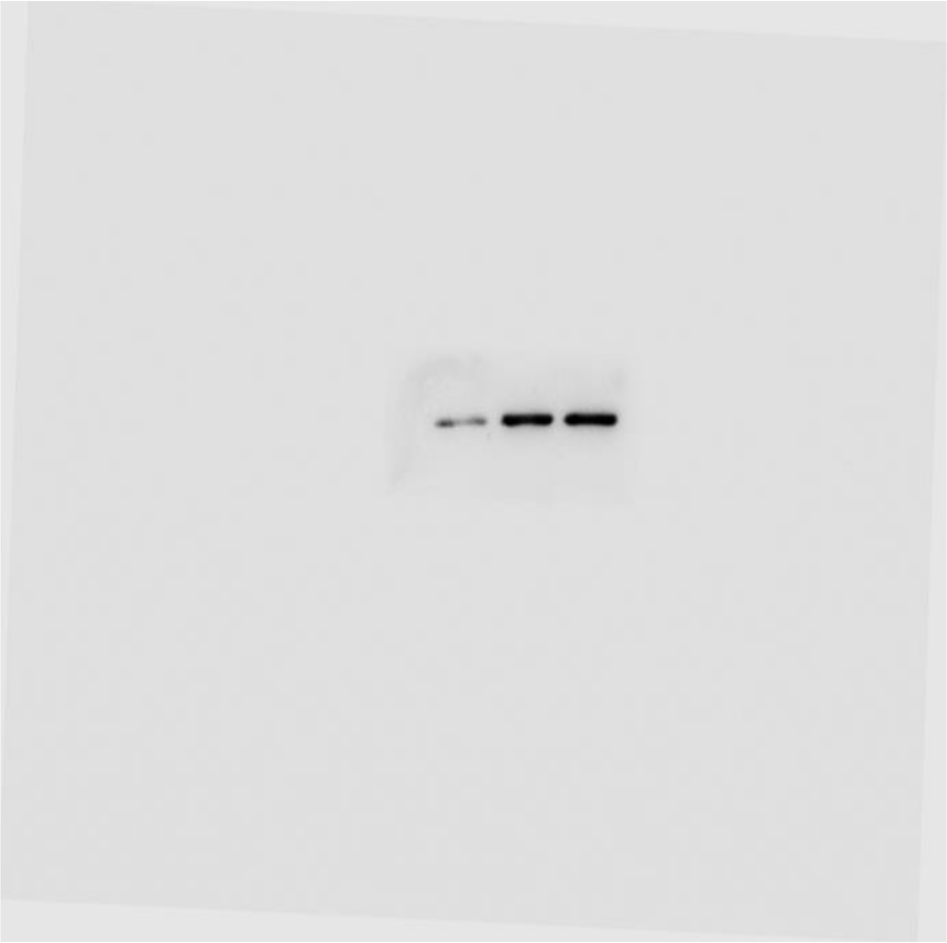

Fig 6I

IB: PGC-1α (SNU-423)

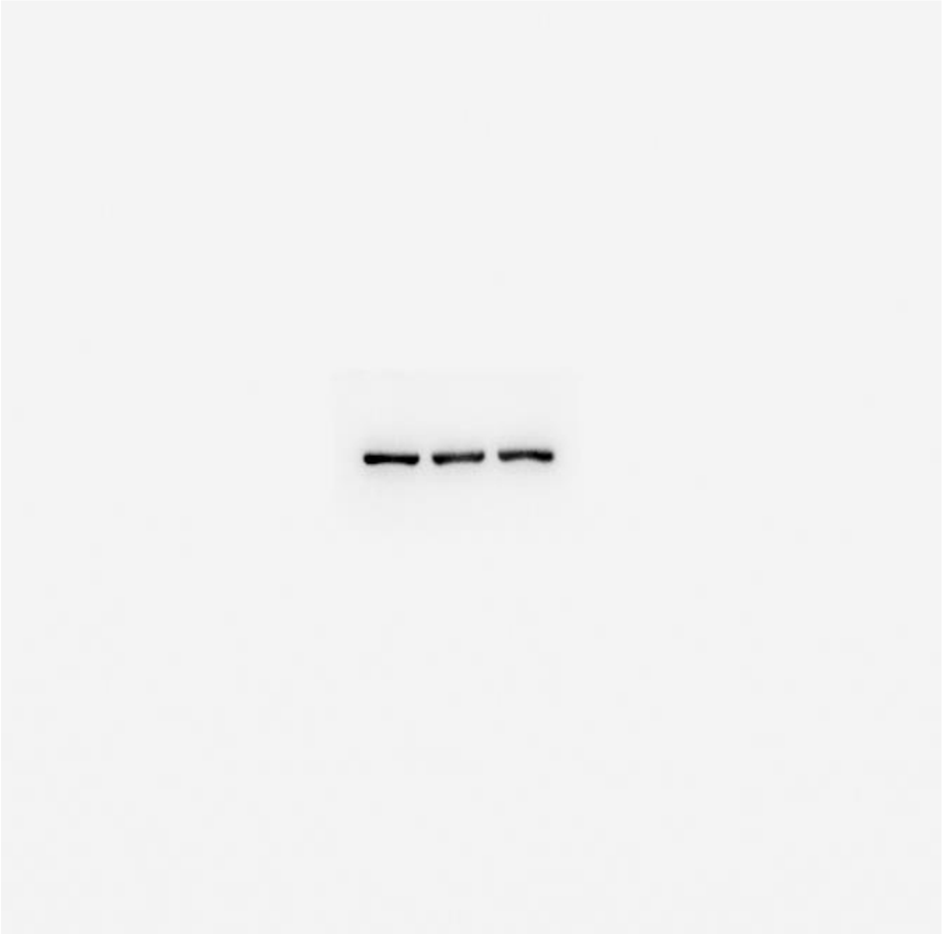

Fig 6I

IB: AC (SNU-449)

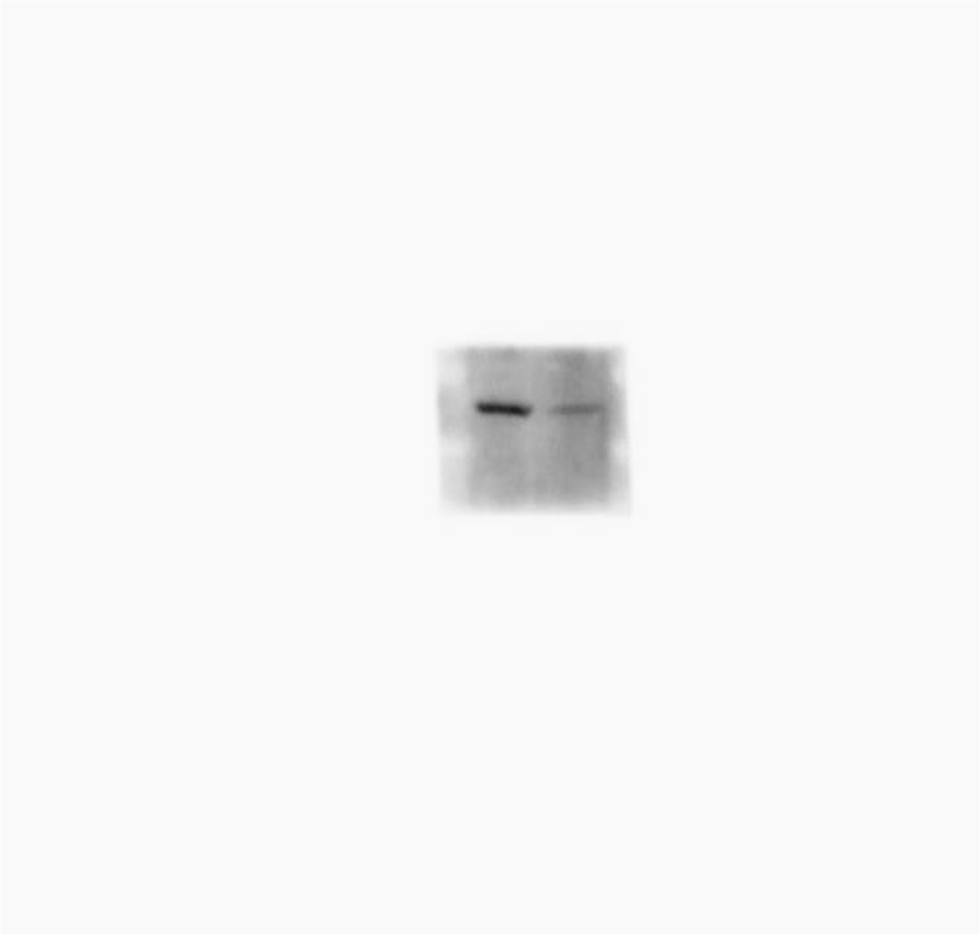

Fig 6I

IB: PGC-1α (SNU-449)

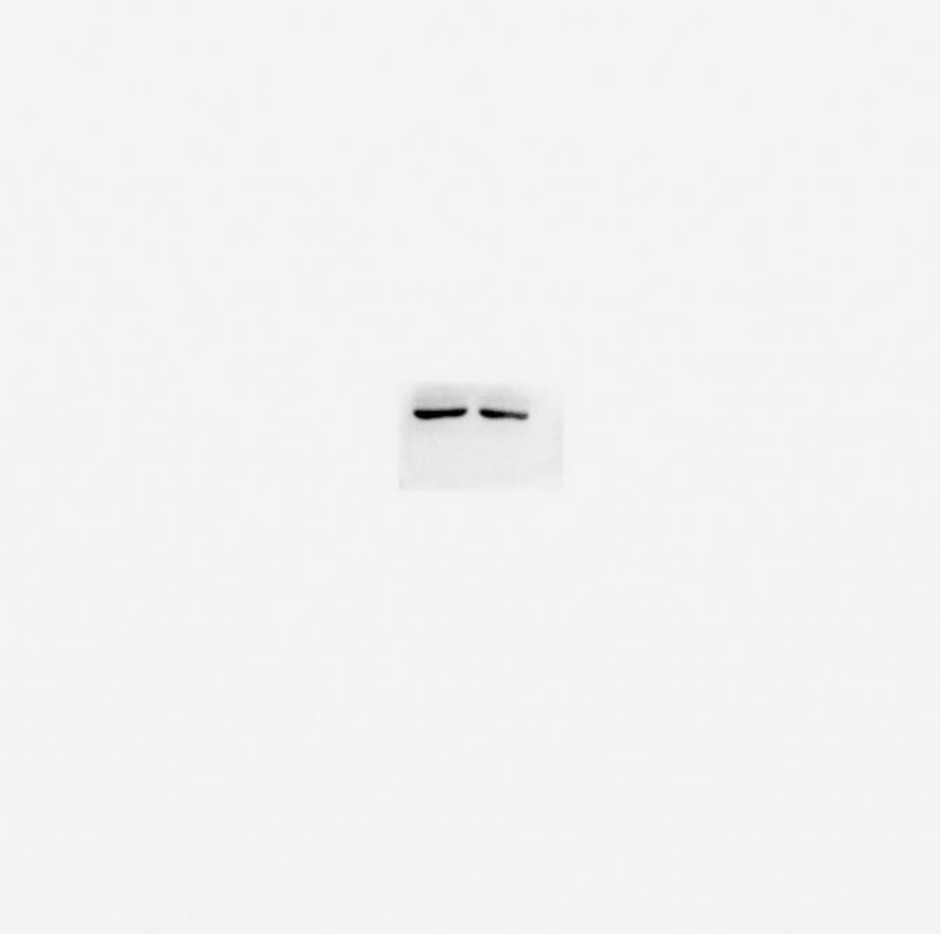

Fig 6J

PGC-1α (SNU-423)

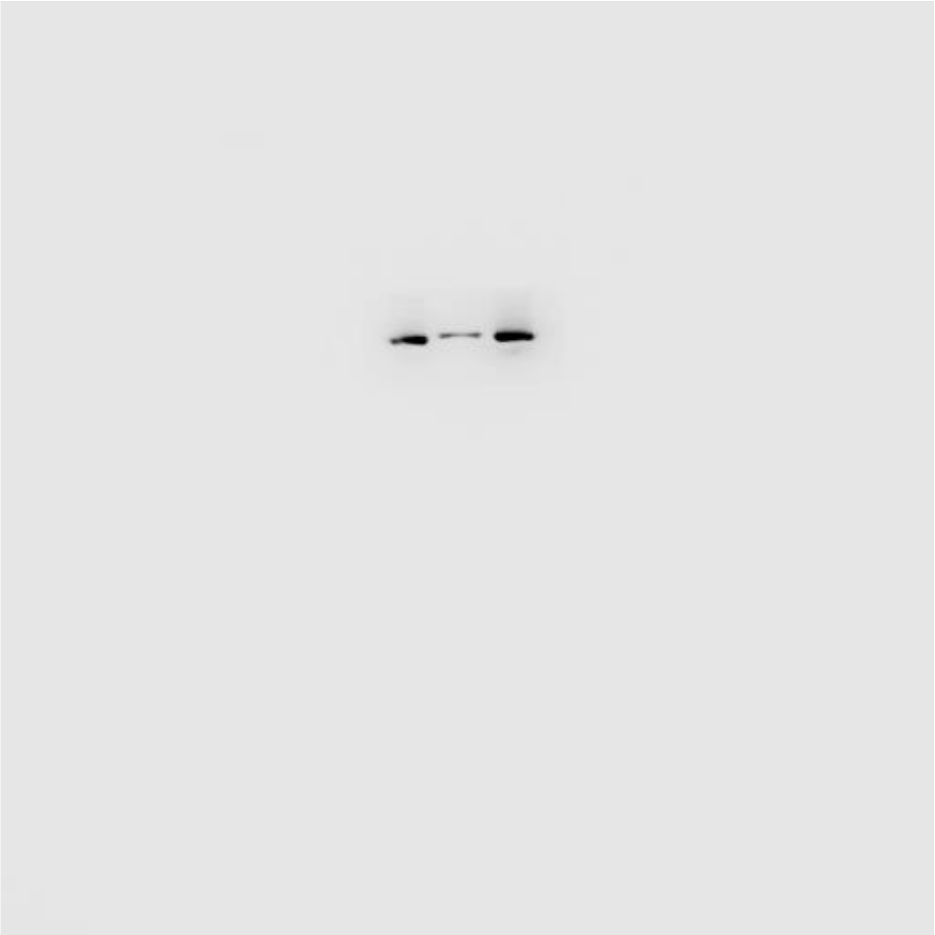

Fig 6J

β-actin (SNU-423)

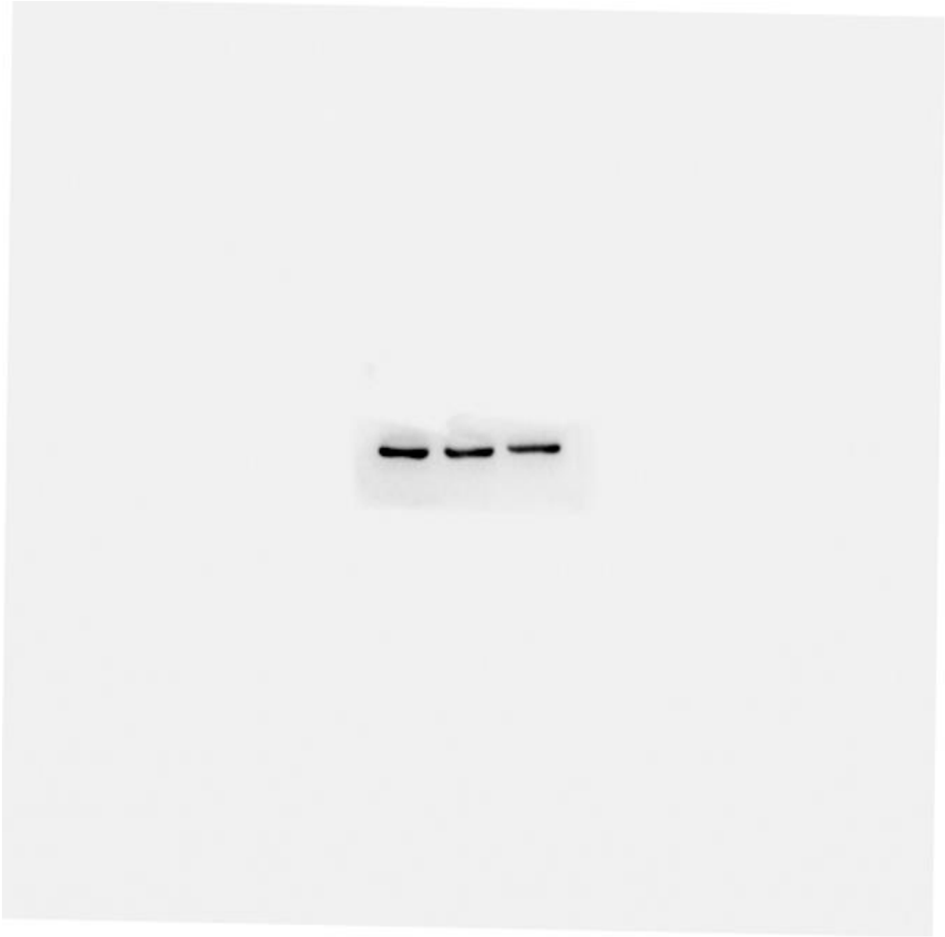

Fig 6J

PGC-1α (SNU-423)

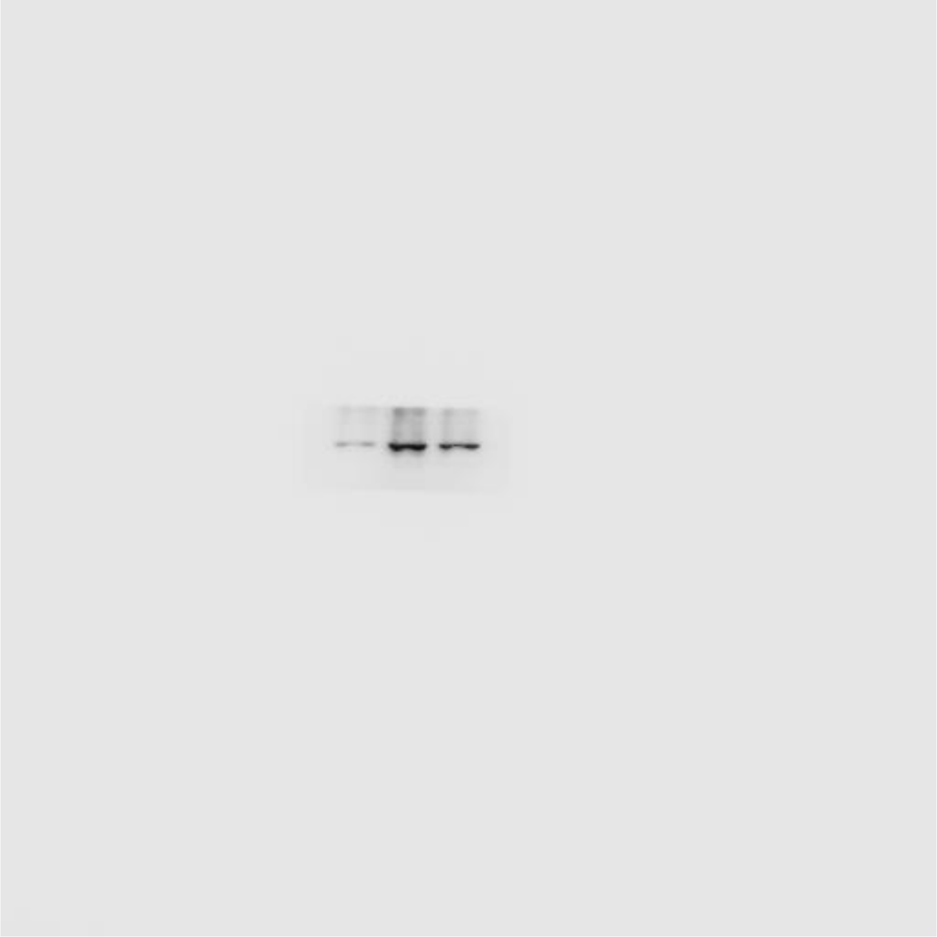

Fig 6J

β-actin (SNU-449)

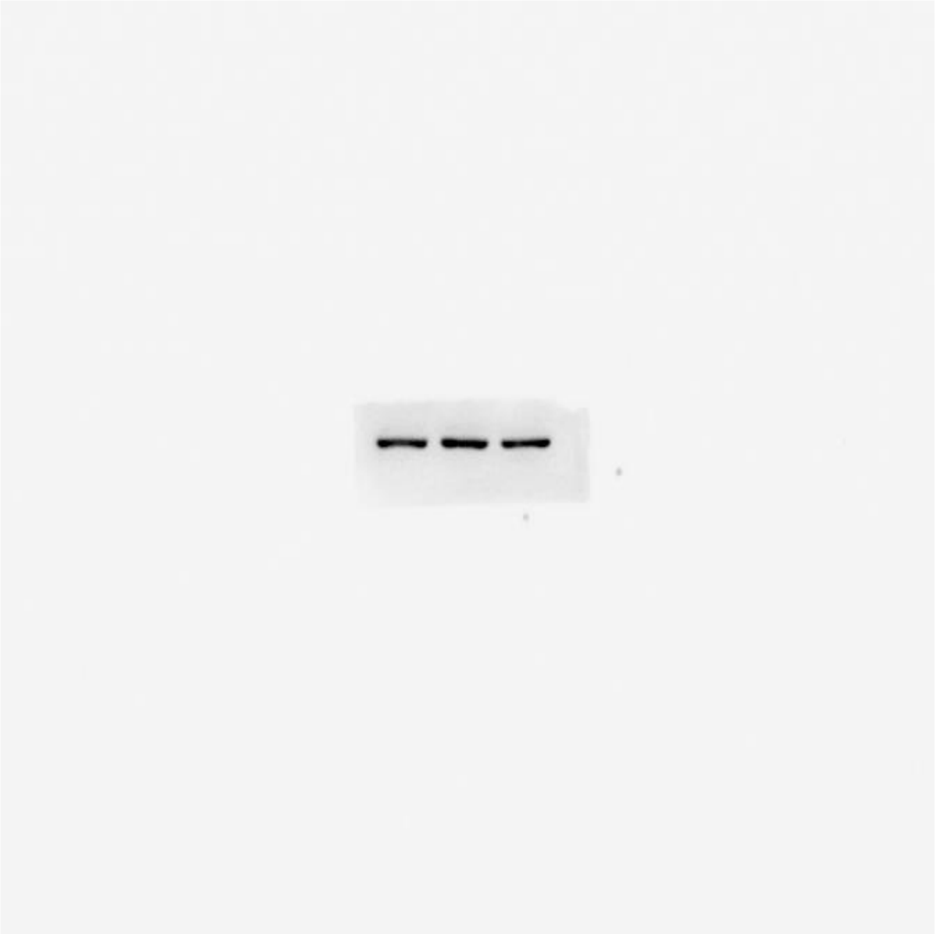

Fig 7B

AIFM2 (SNU-423)

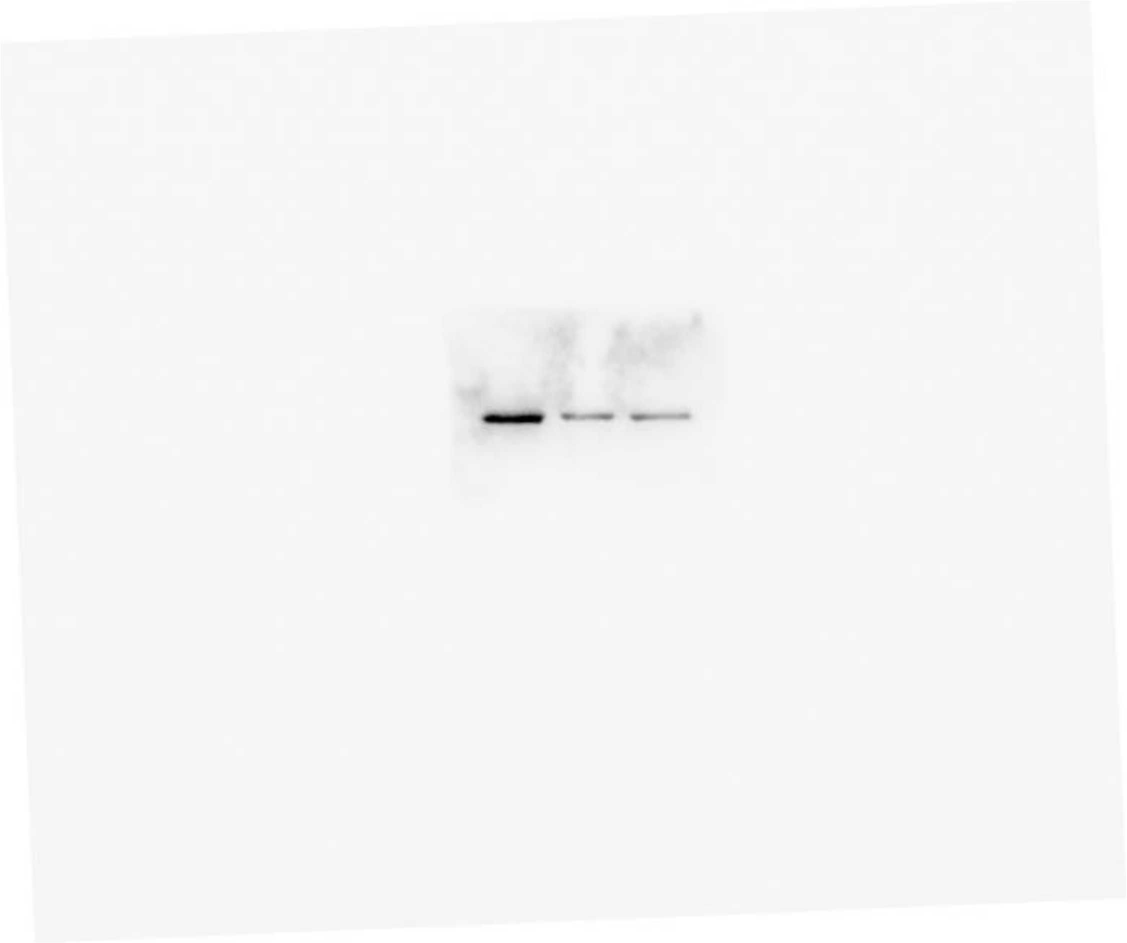

Fig 7B

PGC-1α (SNU-423)

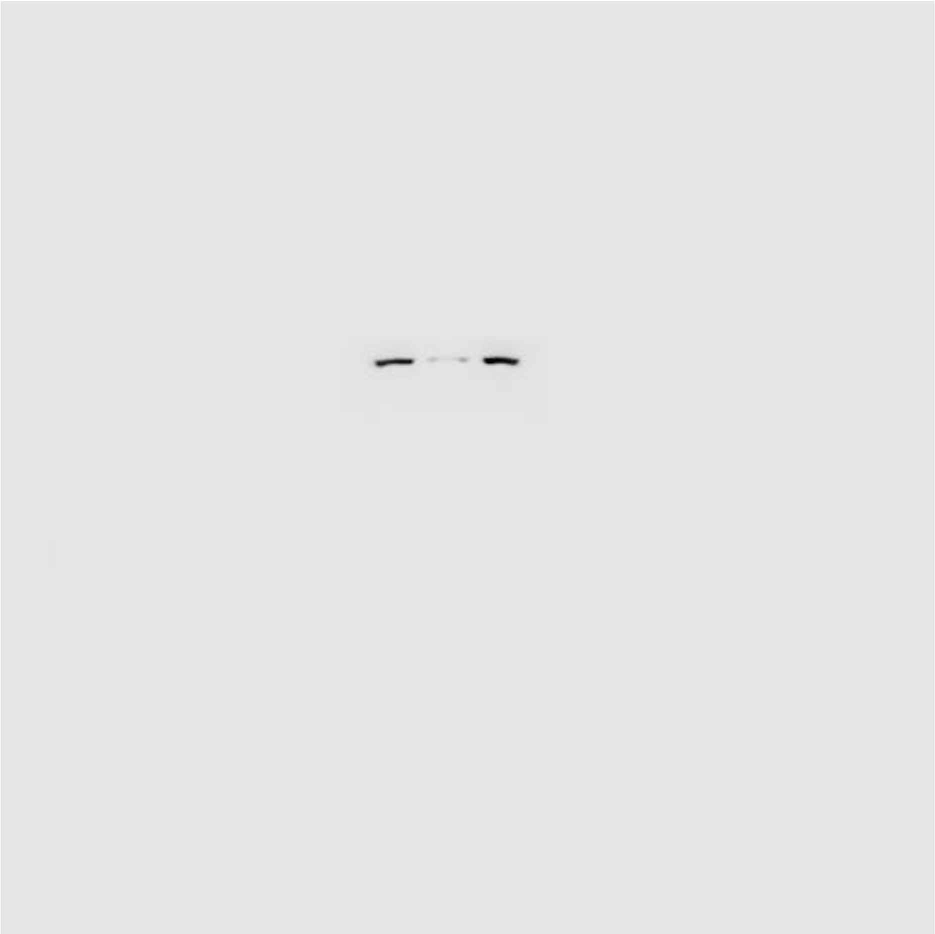

Fig 7B

β-actin (SNU-423)

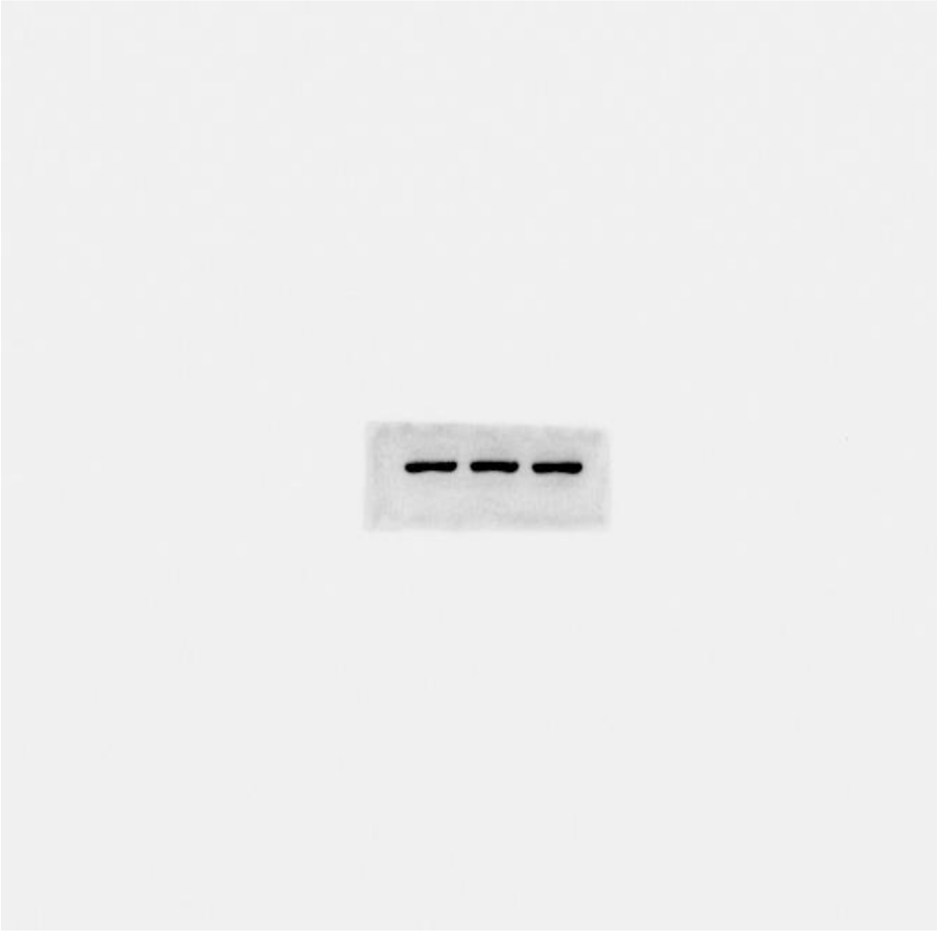

Fig 7B

AIFM2 (SNU-449)

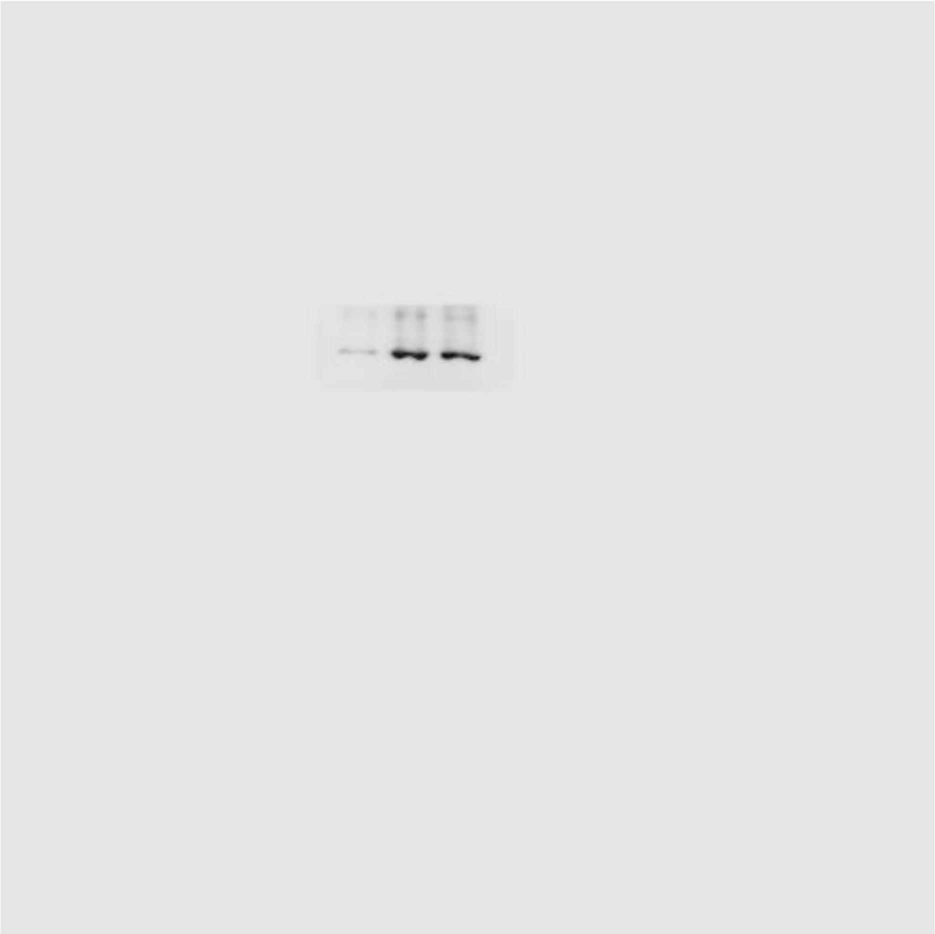

Fig 7B

PGC-1α (SNU-449)

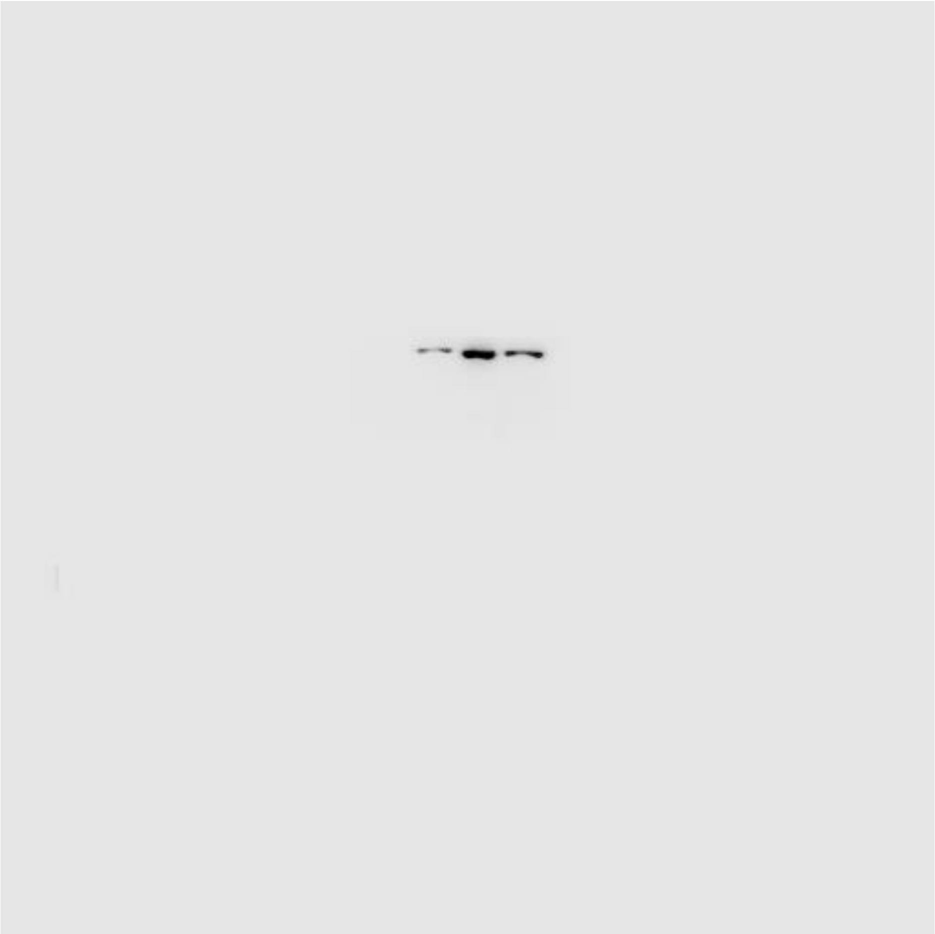

Fig 7B

β-actin (SNU-449)

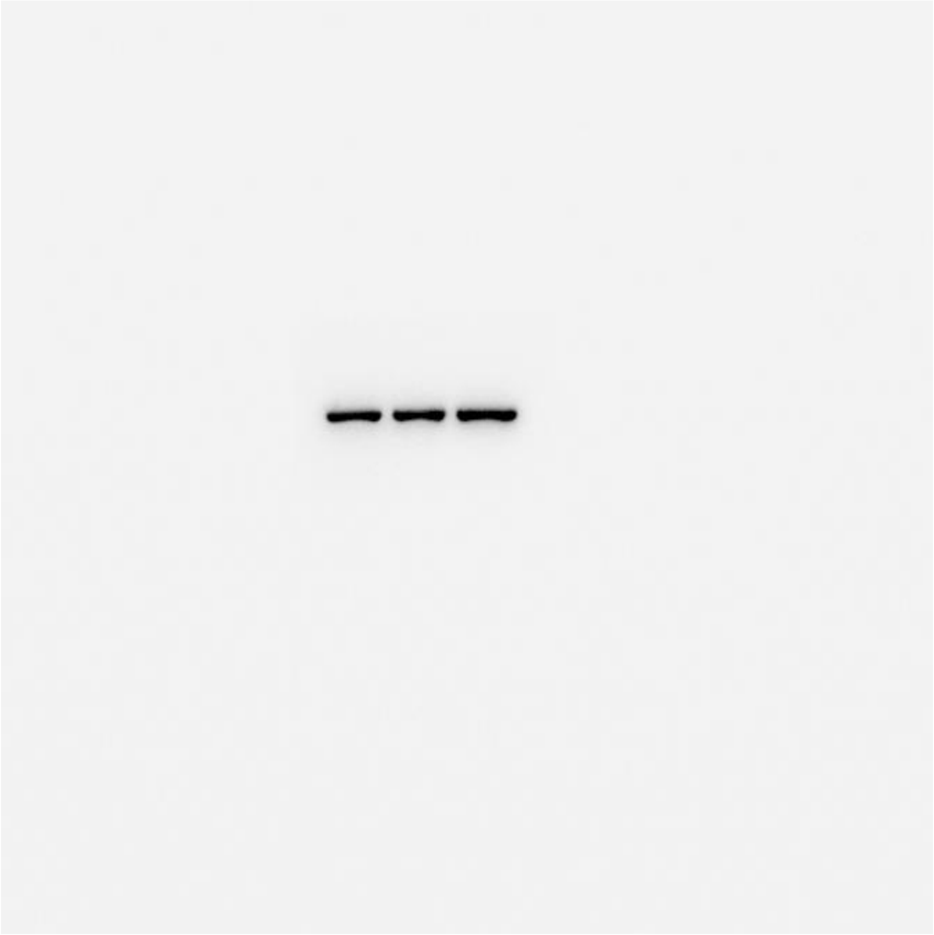

Supplement: Supplementary file 2 — Original Data File [file 41389_2023_491_MOESM2_ESM.pdf]
